# Transcriptomic profiles of *Dunaliella salina in* response to hypersaline stress

**DOI:** 10.1186/s12864-020-6507-2

**Published:** 2020-02-03

**Authors:** Qinghua He, Yaqiu Lin, Hong Tan, Yu Zhou, Yongli Wen, Jiajia Gan, Ruiwen Li, Qinglian Zhang

**Affiliations:** 1 Key Laboratory of Qinghai-Tibetan Plateau Animal genetic Resource Reservation and Utilization, College of Life Science and Technology, Southwest Minzu University, Chengdu, People’s Republic of China; 2 Institute of Qinghai-Tibetan Plateau, Southwest Minzu University, Chengdu, People’s Republic of China; 3 Reproductive and endocrine laboratory, Chengdu Woman-Child Central Hospital, Chengdu, 610051 People’s Republic of China; 4 School of Laboratory Medicine, Chengdu Medical College, Chengdu, 610500 People’s Republic of China

**Keywords:** *Dunaliella salina*, Salt stress, Glycerol, Transcriptomics analysis, Third-generation sequencing, Second-generation sequencing

## Abstract

**Background:**

*Dunaliella salina* is a good model organism for studying salt stress. In order to have a global understanding of the expression profiles of *Dunaliella salina* in response to hypersaline stress, we performed quantitative transcriptomic analysis of *Dunaliella salina* under hypersaline stress (2.5 M NaCl) of different time duration by the second and third generation sequencing method.

**Results:**

Functional enrichment of the up-regulated genes was used to analyze the expression profiles. The enrichment of photosynthesis was observed, accompanied by enrichments of carbon fixation, pigment biosynthetic process and heme biosynthetic process, which also imply the enhancement of photosynthesis. Genes responsible for starch hydrolysis and glycerol synthesis were significantly up-regulated. The enrichment of biosynthesis of unsaturated fatty acids implies the plasma membrane undergoes changes in desaturation pattern. The enrichment of endocytosis implies the degradation of plasma membrane and might help the synthesis of new glycerophospholipid with unsaturated fatty acids. Co-enrichments of protein synthesis and degradation imply a higher protein turnover rate. The enrichments of spliceosome and protein processing in endoplasmic reticulum imply the enhancement of regulations at post-transcriptional and post-translational level. No up-regulation of any Na^+^ or Cl^−^ channels or transporters was detected, which implies that the extra exclusion of the ions by membrane transporters is possibly not needed. Voltage gated Na^+^ and Cl^−^ channels, mechanosensitive ion channel are possible signal receptors of salt stress, and Ca^2+^ and MAP kinase pathways might play a role in signal transduction.

**Conclusion:**

At global transcriptomic level, the response of *Dunaliella salina* to hypersaline stress is a systematic work, possibly involving enhancements of photosynthesis, carbon fixation, and heme biosynthetic process, acceleration of protein turnover, spliceosome, protein processing in endoplasmic reticulum, and endocytosis, as well as degradation of starch, synthesis of glycerol, membrane lipid desaturation. Altogether, the changes of these biological processes occurred at trancriptomic level will help understand how a new intracellular balance achieved in *Dunaliella salina* to adapt to hypersaline environment, which are worth being confirmed at the physiological levels.

## Background

*Dunaliella* is an extremely halotolerant, unicellular, green algae, which is unique in its remarkable ability to survive in media containing NaCl at a wide range of concentrations, from about 0.05 M to saturation (around 5.5 M) [1]. This character makes it a good model organism for studying salt tolerance. Studies on salt tolerance of *Dunaliella* began from 60s last century, and big progresses were made from 70s to 90s. First, high concentration of intracellular glycerol was found to be the main contributor for osmotic balance across plasma membrane [2]. Second, a glycerol metabolism cycle in *Dunaliella* was proposed, that is, for glycerol synthesis, dihydroxyacetone phosphate (DHAP) from glycolysis is converted to glycerol-3-phosphate by glycerol-3-phosphate dehydrogenase (GPDH), then gycerol-3-phosphate is converted to glycerol by glycerol-3-phosphate phosphatase; and for glycerol dissimilation, glycerol is converted to dihydroxyacetone by glycerol dehydrogenase, and then dihydroxyacetone is converted to DHAP by dihydroxyacetone kinase [3]. As the key enzyme in the pathway, GPDH was extensively studied [4, 5]. Third, the Na^+^/H^+^ antiporter activity was detected in plasma membrane and was thought to function as exclusion of Na^+^ in vivo [6, 7].

In twenty-first century, proteomic methods were used to understand the molecular mechanism of salt tolerance at omics level. Proteins such as transferrin, carbonic anhydrases, Na^+^/H^+^ antiporter, fatty acid elongase, GPDH, small GTP-binding protein and tubulin were found up-regulated significantly under salt stress. These proteins can be classified in carbon assimilation, energy production, transporters, signal transduction, protein synthesis and cell defense [8, 9]. However, due to the limitation of the two-dimensional electrophoresis, the information obtained from this technique is limited [8, 9], with detected number of differently expressed proteins below 100, of which only about 60% can be annotated.

Compared with proteomic approaches, transcriptomic methods are more reproducible, sensitive with higher genome coverage. A transcrtiptome of 17,845 transcripts was reported when *Dunaliella tertiolecta* was investigated to identify the pathways and genes involved in lipid synthesis under nitrogen stress, which covers about 97% of the core eukaryotic genes (CEGs) [10–12]. Hong et al. reported the transcriptome of *Dunaliella salina* at different phases of their growth cycle (30d, 80d, 120d), but no transcriptome under salt stress was reported [13]. Alkayal reported the expressed sequence tag (EST) profiling of *Dunaliella salina* after 5 h of hypersaline shock, in which a transcriptome of 1401 unique transcripts was reported and the annotated transcripts can be classified into protein synthesis, energy, primary metabolism and protein fate [14]. However, no transcriptome before salt stress was generated, so there was no comparison to present the underlying changes during this shock period. In order to have a better understanding of how *Dunaliella salina* responds to hypersaline shock at transcriptomic level, the second and the third generation sequencing were used to generate the transcriptome of *Dunaliella salina* at different duration time under stress. Because intracellular glycerol synthesis accomplished in about 2 h after hypersaline shock [15, 16], we reported the transcriptomic profiles on time duration of 0.5-h, 1-h and 2-h under hypersaline stress and the profiles were compared with those before stress.

## Results

### Data quality and sequences annotation

To obtain high quality sequence data, total RNAs of high quality were extracted (not shown). After the second generation sequencing, each library gave high quality clean reads with Q20% ranging from 97.21 to 98 with error rate about 0.01% (Additional file 1: Table S1). The GC content is about 56.5%, which is close to *Dunaliella salina* (CCAP19/18) reported [17] . The number of the named “full length transcripts” generated from the third generation sequencing was 43,864, ranging from 242 to 8978 bp in length with a mean length of 1009 bp and median length of 918 bp. About 80% transcripts of them are in the length range of 500 to 2000 bp (Additional file 1: Table S2). By ORF analysis, among the 43,864 transcripts, 35,175 transcripts are classified into coding sequences and 8689 transcripts are classified into long non-coding sequences. Among the 35,175 coding sequences, 29,071 sequences are annotated and 6104 sequences cannot be annotated so far. In order to estimate the coverage of the transcriptome, transcripts hit the same gene (the same sequence ID) in Nr, Nt or SwissProt database are defined as the splice variants generated by alternative splicing from a single gene. By this method isoenzymes and artificially spliced sequences are also excluded. Finally 9256 individual genes from the 29,071 transcripts are generated. Genome sequencing of *Dunaliella salina* (CCAP19/18) and *Chlamydomonas reinhardtii* predicted 16,697 and 17,741 loci containing protein-coding transcripts respectively [17, 18]. Compared with the predicted numbers of gene loci of the two green algae, 9256 is a rather high number, since many genes aren’t expressed and their mRNAs can’t be detected. Furthermore, approximately 87.1% of the core eukaryotic genes (CEGs) were identified from the 9256 individual genes by sequence similarity search which suggests a rather high coverage of the *Dunaliella salina* transcriptome.

### General pattern of gene expression

Based on gene expression value, clustering analysis was performed (Additional file 2: Figure S1), we can see the similarities of the expression patterns of the samples with good repeatability in the same group (the same stress time).

**Fig. 1:**
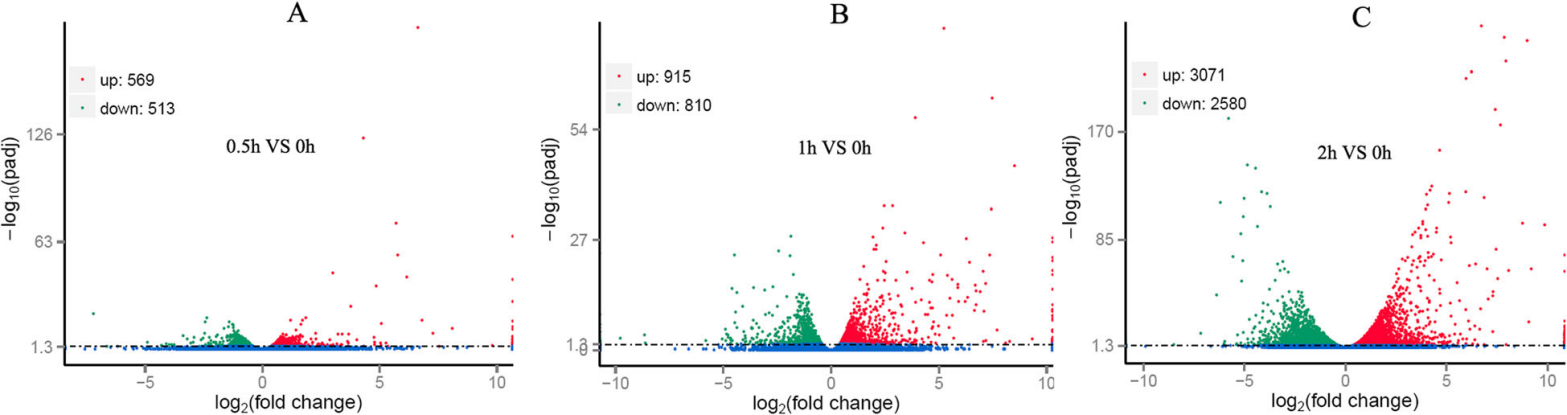
Volcano Plot of the differentially expressed genes. The differentially expressed genes were generated by comparing the gene expression values under stress of different time duration (0.5 h, 1 h, 2 h) with that of control (0 h). **a** the comparison of 0.5-h of stress with that of 0-h of stress; **b** the comparison of 1-h of stress with that of 0-h of stress; **c** the comparison of 2-h of stress with that of 0-h of stress; the number of up-regulated genes increased constantly with the increasing of stress duration time, the number of down-regulated genes also increased constantly with the increasing of stress duration time

While compared with the 0-h of stress (no salt stress was applied), the number of differentially expressed genes increased with the increasing of stress duration time (Fig. 1). The number of up-regulated genes increases from 569 on 0.5-h of stress to 915 on 1-h of stress, and then to 3071 on 2-h of stress. On the other hand, the number of down-regulated genes increases from 513 on 0.5-h stress to 810 on 1-h stress, and then to 2580 on 2-h stress.

In order to have an overall understanding of the up-regulated genes under salt stress, functional enrichments were performed by GO (gene ontology) (Table 1). On 0.5-h of stress, carboxylic acid biosynthetic process, cellular lipid metabolic process, carbohydrate metabolic process, response to temperature stimulus, photosynthesis (light harvesting), photosynthesis (light reaction), cofactor metabolic process, pigment biosynthetic process, and tetrapyrrole biosynthetic process are significantly enriched. On 1-h of stress, protein folding and DNA replication are included in the list of significantly enriched biological processes, cellular lipid metabolic process and response to temperature stimulus are enriched but not statistically significant, while photosynthesis is excluded due to rapid decreasing of gene number (Table 2). On 2-h of stress, new terms such as macromolecule modification, cellular catabolic process, cell redox homeostasis, reproductive process, and ferrous iron transport are significantly enriched, while transcription (DNA-templated) is enriched, but not statistically significant. The terms enriched on 1-h of stress, such as carboxylic acid metabolic process, cellular lipid metabolic process, carbohydrate metabolic process, response to temperature stimulus, cofactor metabolic process, protein folding, and DNA replication, are also enriched and show a quick increasing of the gene numbers compared with that of 1-h of stress. These biological processes are not statistically significant due to the rapid increasing of the number of the total up-regulated genes, but they are still worth focusing on. In general, the significantly enriched biological processes can be classified into photosynthesis, carbohydrate metabolism, lipid metabolism, and amino acids and protein metabolism. We focused on analyzing these biological processes in the following sections.

**Table 1:** Main biological processes significant enriched from the up-regulated genes

GO_accession	Description	Number of Genes involved
0.5 h VS 0 h		
GO:0046394	carboxylic acid biosynthetic process	29
GO:0044255	cellular lipid metabolic process	26
GO:0005975	carbohydrate metabolic process	44
GO:0009266	response to temperature stimulus	12
GO:0009765	photosynthesis, light harvesting	35
GO:0019684	photosynthesis, light reaction	38
GO:0051186	cofactor metabolic process	52
GO:0046148	pigment biosynthetic process	24
GO:0033014	tetrapyrrole biosynthetic process	27
1 h VS 0 h		
GO:0019752	carboxylic acid metabolic process	100
GO:0044255	cellular lipid metabolic process^a^	36
GO:0005975	carbohydrate metabolic process	73
GO:0009266	response to temperature stimulus^a^	14
GO:0051186	cofactor metabolic process	74
GO:0046148	pigment biosynthetic process	28
GO:0033014	tetrapyrrole biosynthetic process	31
GO:0006457	protein folding	26
GO:0006260	DNA replication	28
2 h VS 0 h		
GO:0019752	carboxylic acid metabolic process^a^	230
GO:0044255	cellular lipid metabolic process^a^	61
GO:0005975	carbohydrate metabolic process	133
GO:0009266	response to temperature stimulus^a^	23
GO:0051186	cofactor metabolic process^a^	127
GO:0006457	protein folding	48
GO:0006260	DNA replication^a^	57
GO:0006351	transcription, DNA-templated^a^	191
GO:0043412	macromolecule modification	134
GO:0044248	cellular catabolic process	79
GO:0045454	cell redox homeostasis	32
GO:0022414	reproductive process	30
GO:0015684	ferrous iron transport	13

^a^not significantly enriched

**Table 2:** Enrichment of photosynthesis and photosynthetic pigments related terms

GO_ID	GO_Term	Samples		
		0.5 h	1 h	2 h
		Number of Genes involved	Number of Genes involved	Number of Genes involved
GO:0015979	photosynthesis	50	40	31
GO:0009765	photosynthesis, light harvesting	35^a^	18	0
GO:0019684	photosynthesis, light reaction	38^a^	21	6
GO:0033014	tetrapyrrole biosynthetic process	27^a^	31^a^	27
GO:0015995	chlorophyll biosynthetic process	9	8	2
GO:0006783	heme biosynthetic process	11^a^	15^a^	22
GO:0046148	pigment biosynthetic process	28^a^	33^a^	48
KO_ID	KO_term	Number of Genes involved	Number of Genes involved	Number of Genes involved
ko00710	carbon fixation in photosynthetic organisms	12	25	39

^a^indicates significantly enriched

On the other hand, the functional enrichment of the down-regulated genes was also performed by GO (Additional file 1: Table S3). On 0.5-h of stress, no terms were significantly enriched, but carbohydrate binding and protein binding were worth focusing on since the numbers of down-regulated genes involved are large. On 1-h of stress, DNA metabolic process, protein binding, cytoskeleton, glycoprotein biosynthetic process, glycosaminoglycan biosynthetic process, and dynein complex were significantly enriched. On 2-h of stress, more GO terms were significantly enriched beside the terms enriched on 1-h of stress, these terms include transferase activity, protein modification process, regulation of RNA biosynthetic process, response to nitrate, inorganic anion transport, lipid transport, DNA integration, autophagy, and GTPase activator activity. From the point of gene numbers, we can see that the down-regulated genes are mainly involved in protein binding, transferase activity, protein modification process, DNA metabolic process, regulation of RNA biosynthetic process, and cytoskeleton. These terms are also important for understanding the hypersaline stress of *Dunaliella salina*, however, this paper only focuses on the analysis of the terms enriched by the up-regulated genes.

### Photosynthesis

**Fig. 2:**
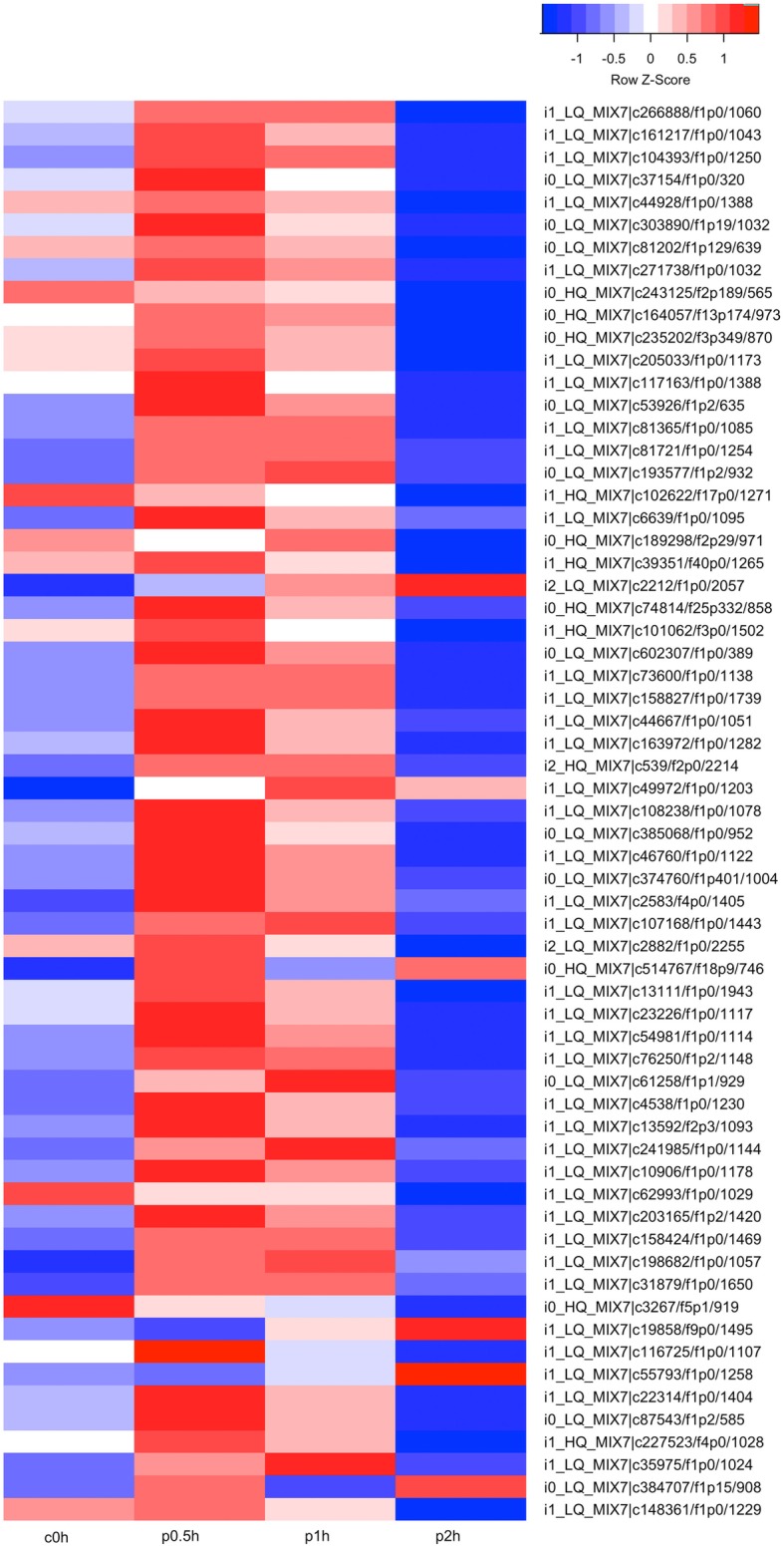
Heat-map of photosynthesis; the colors from blue to red represent the gene express values from low to high. The z-scores represent gene expression values were generated from their FPKMs. The four columns represent the four experimental groups. C0h represents the control group with no hypersaline stress applied. p0.5h, p1 h, and p2 h represent the three hypersaline treated groups with 0.5-h, 1-h, and 2-h time duration. Genes IDs are on the right. Genes are also grouped base on their expression patterns

On the 0.5-h of stress, photosynthesis-light reaction and photosynthesis-light harvesting are significantly enriched by GO analysis on up-regulated genes, which implies the enhancement of photosynthesis. In time course, most genes are highly expressed on 0.5-h, decreased a little on 1-h, and then decreased to low levels even lower than that of 0-h. The expression pattern is like a pulse style and most peaks of gene expression are induced on or before 0.5-h of stress (Fig. 2). Many genes of Chlorophyll a-b binding proteins show pulse expression patterns, such as Chlorophyll a-b binding protein of LHCII type I, Chlorophyll a-b binding protein type 1 member F3, Chlorophyll a-b binding protein P4, and Chlorophyll a-b binding protein CP29 et al.. Some of the genes show high expression on 2-h of stress, including ATP-dependent zinc metalloprotease FTSH 2, Photosystem II repair protein PSB27-H1, D-amino-acid transaminase, and Photosystem II protein D1. A few genes show a decreasing of expression, including Protein TIC 20-II, Oxygen-evolving enhancer protein, and DNA-binding 11 kDa phosphoprotein (Fig. 2). Chlorophyll biosynthetic process is also enriched, which indicates the synthesis of photosynthetic pigments to enhance photosynthesis (Table 2). This is consistent with previous study [9]. With the stress going on, the gene numbers of photosynthesis-light reaction and photosynthesis-light harvesting decreased (Table 2), while the gene number of carbon fixation constantly increased, from 12 on 0.5-h to 25 on 1-h, and to 39 on 2-h of stress (Table 2, Additional file 2: Figure S2), key genes such as carbonic anhydrase and rubisco activase are significantly up-regulated (Additional file 1: Table S4). Compared with the decreased gene number of photosynthesis-light reaction and photosynthesis-light harvesting, the constantly increased gene number of carbon fixation indicates that these biological processes may be controlled by different signaling pathways.

With the stress going on, the gene number of chlorophyll biosynthetic process decreased, while the gene number of tetrapyrrole biosynthetic process remained stable and the gene number of heme biosynthetic process kept increasing (Table 2). The increasing of gene number of heme biosynthetic process and the decreasing of gene number of chlorophyll biosynthetic process together resulted in the stableness of gene number of tetrapyrrole biosynthetic process since the latter is the father term of the former two. This is consistent with the result of heat-map analysis, of which some genes show pulse expression pattern, these genes are clustered to chlorophyll biosynthetic process, while some genes show high expression values on 2-h of stress, these genes are clustered to heme biosynthetic process (Additional file 2: Figure S3). The significant enrichment of tetrapyrrole biosynthetic process and heme biosynthetic process on 0.5-h and 1-h of stress are very interesting. In plants and algae, tetrapyrroles are plastid signals demonstrated to regulate nuclear gene expression [19–22]. Heme signaling also appears to play a role in starch biosynthesis and drought tolerance in plants [23, 24]. We see the constant increasing of gene number of heme biosynthetic process with the increasing of stress time, while large amount of signal molecules are usually not needed, so the constant increasing gene number of heme synthesis could be for the synthesis of heme-containing enzymes, such as catalase and ascorbate peroxidase, which play important roles in detoxification of reactive oxygen species (ROS) [25]. Consistently, the expression of ascorbate peroxidase is up-regulated and also confirmed by qPCR (Additional file 1: Table S4, Additional file 3).

### Starch and sucrose metabolism

**Fig. 3:**
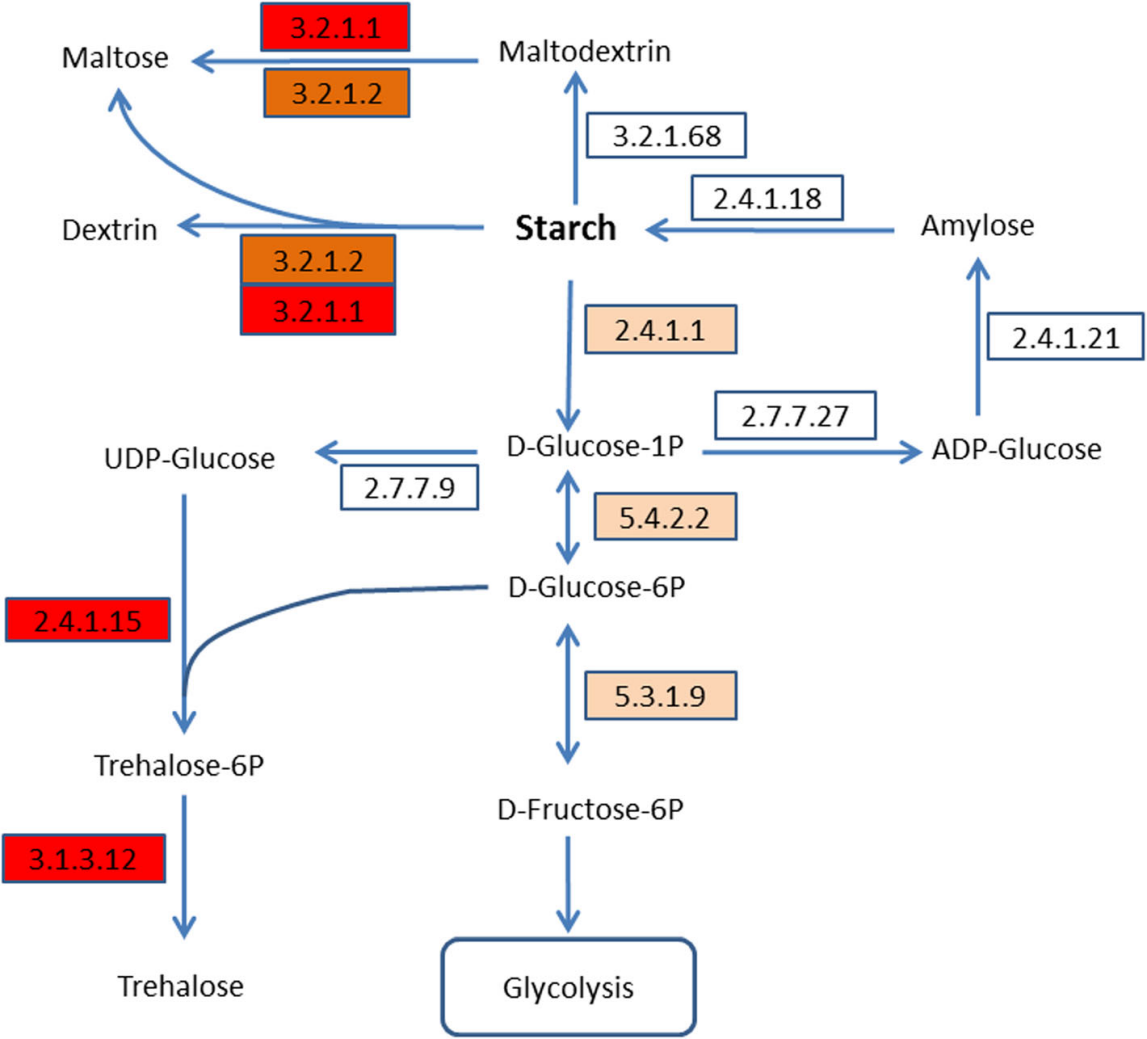
The simplified pathway of starch metabolism. The numbers in the rectangles are enzyme codes, all the enzymes are identified in the transcriptome, the arrows show the direction of enzyme-catalyzed reaction; enzymes up-regulated on 0.5-h of stress are highlighted by light orange; enzymes up-regulated on 1-h of stress are highlighted by orange, enzymes up-regulated on 0.5-h of stress were also up-regulated on 1-h of stress; enzymes up-regulated on 2-h of stress are highlighted by red, enzymes up-regulated on 0.5-h and 1-h of stress were also up-regulated on 2-h of stress

Starch and sucrose metabolism is significantly enriched by KEGG Pathway analysis on up-regulated genes. On 0.5-h of stress, the expression of starch phosphorylase (PYG, 2.4.1.1), which catalyzes the hydrolysis of starch into alpha-D-glucose 1-phosphate, is significantly up-regulated (Additional file 1: Table S4). At the same time, the expression of phosphoglucomutase (PGM, 5.4.2.2, catalyzing alpha-D-glucose 1-phosphate to alpha-D-glucose 6-phosphate) and glucose-6-phosphate isomerase (GPI, 5.3.1.9, catalyzing alpha-D-glucose 6-phosphate to beta-D-fructose-6-phosphate) are significantly up-regulated (Additional file 1: Table S4), implying the alpha-D-glucose 1-phosphate from starch hydrolysis may go into glycolysis pathway (Fig. 3). On 1-h of stress, beta-fructofuranosidase (3.2.1.26, not shown on Fig. 3) and beta-amylase (3.2.1.2) are significantly up-regulated. On 2-h of stress, alpha-amylase (3.2.1.1), trehalose 6-phosphate synthase (otsA, 2.4.1.15) and trehalose 6-phosphate phosphatase (otsB, 3.1.3.12) are significantly up-regulated (Fig. 3).

On the whole, genes catalyzing the hydrolysis of polysaccharide (such as starch and maltodextrin) and disaccharide (such as sucrose and maltose) are significantly up-regulated (Table 3). Other up-regulated genes besides polysaccharide hydrolysis, include trehalose 6-phosphate synthase (otsA, 2.4.1.15) and trehalose 6-phosphate phosphatase (otsB, 3.1.3.12) (Table 3). The up-regulation of otsA and otsB synchronously indicates the accumulating of trehalose (Fig. 3), which is not a reducing sugar and reported to play a role in abiotic stress tolerance [26]. The existing of PYG, alpha-amylase, beta-amylase, isoamylase (ISA, 3.2.1.68), and cyclomaltodextrin glucanotransferase (cgt, EC: 2.4.1.19, not shown on Fig. 3) indicates that there are alternative pathways for starch hydrolysis.

**Table 3:** Up-regulated enzymes involved in starch and sucrose metabolism

Enzyme code	Name	Reaction
Polysaccharide degradation		
2.4.1.1	Glycogen phosphorylase	[(1- > 4)-alpha-D-glucosyl] n + phosphate = [(1- > 4)-alpha-D-glucosyl]n-1 + alpha-D-glucose 1-phosphate
3.2.1.1	alpha-amylase	Starch + H2O < => Dextrin + Starch
3.2.1.2	beta-amylase	Starch <= > Dextrin + Maltose
Disaccharide degradation		
3.2.1.26	beta-fructofuranosidase	Sucrose + H2O < => D-Fructose + D-Glucose
2.4.1.25	4-alpha-glucanotransferase	Amylose + n D-Glucose <= > n Maltose
Others		
2.4.1.15	trehalose 6-phosphate synthase	UDP-glucose + D-Glucose 6-phosphate <= > UDP + alpha,alpha’-Trehalose 6-phosphate
3.1.3.12	trehalose 6-phosphate phosphatase	alpha,alpha’-Trehalose 6-phosphate + H2O < => alpha,alpha-Trehalose + Orthophosphate

### Glycolysis and glycerol synthesis

**Fig. 4:**
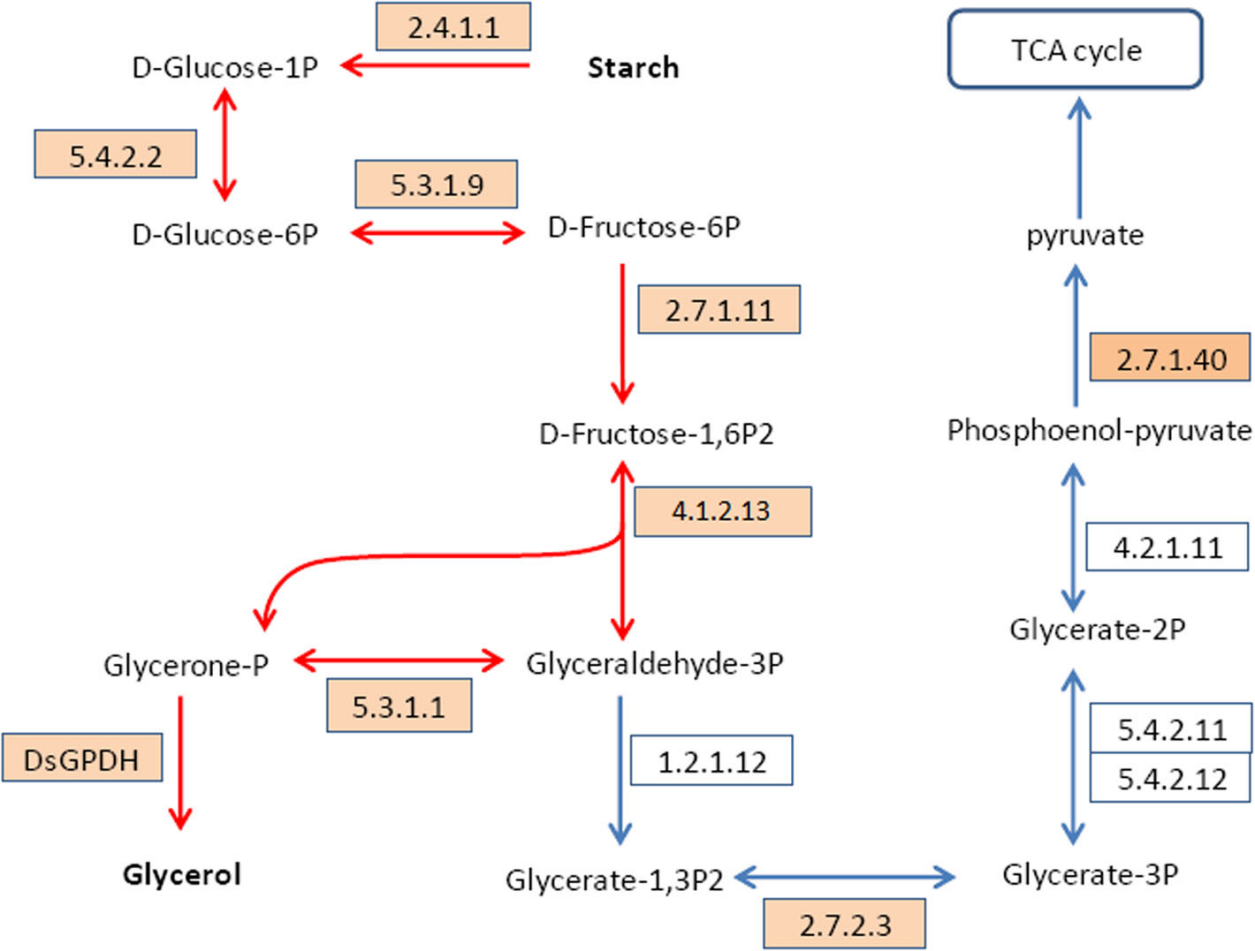
The pathway of glycolysis and glycerol synthesis. The numbers in the rectangles are enzyme codes, all the enzymes are identified in the transcriptome, the arrows show the direction of enzyme-catalyzed reaction, the red arrows show the synthesis of glycerol from starch; enzymes up-regulated on 0.5-h of stress are highlighted by light orange

Glycolysis is significantly enriched by KEGG Pathway analysis on up-regulated genes. The up-regulations of PGM, GPI, the rate-limiting enzyme PFK1 (6-phosphofructokinase 1, 2.7.1.11), and fructose-bisphosphate aldolase were seen on 0.5-h of stress (Additional file 1: Table S4), which implies alphpa-D-Glucose-1p from hydrolysis of starch goes to glycolysis (Fig. 4). Interestingly, triosephosphate isomerase (TPI, 5.3.1.1), which catalyzing the reversible interconversion of Glyceraldehyde 3-phosphate (GADP) and Glycerone phosphate (also known as Dihydroxyacetone phosphate, DHAP), was significantly up-regulated on 0.5-h of stress.

Our data show that the *Dunaliella salina* specific di-domain glycerol-3-phosphate dehydrogenase (DsGPDH) can convert DHAP (an intermediate of glycolysis) to glycerol directly [27]. So the glycerol synthesis pathway of *Dunaliella salina* can be drawn based on the genes from the transcriptomic data (Fig. 4). The new glycerol synthesis pathway includes only 6 enzyme-catalyzing steps to convert starch to glycerol, the six enzymes are all up-regulated on 0.5-h of stress. The up-regulation of TPI can be explained as that more enzyme is needed to converting GADP to DHAP for glycerol synthesis. Consistently, DsGPDH is up-regulated 0.5, 0.9 and 1.0 fold on 0.5-h, 1-h, and 2-h of stress respectively (Additional file 1: Table S4).

### Lipid metabolism

On 0.5-h of stress, fatty acid biosynthesis is significantly enriched by KEGG Pathway analysis on up-regulated genes. The number of genes involved in this pathway remains stable on 1-h of stress, and decreases a little on 2-h of stress. While the gene number of fatty acid degradation and biosynthesis of unsaturated fatty acids increase constantly (Table 4), which implies that fatty acid biosynthesis and degradation occurs synchronously when salt stress is applied. The up-regulated genes of fatty acid biosynthesis include acetyl-CoA carboxylase, long-chain acyl-CoA synthetase, FabZ (3-hydroxyacyl-[acyl-carrier-protein] dehydratase), enoyl-[acyl-carrier-protein] reductase, oleoyl-acyl carrier protein thioesterase, acyl-[acyl-carrier-protein] desaturase, and biotin carboxyl carrier protein of acetyl-CoA carboxylase. However the up-regulation of acetyl-CoA carboxylase wasn’t confirmed by qPCR, but the up-regulation of acetyl-CoA carboxylase beta subunit was confirmed by qPCR (Additional file 3). On 2-h of stress, key genes involved in biosynthesis of unsaturated fatty acids are also significantly up-regulated, including delta-7 desaturase, omega-3 fatty acid desaturase and delta-12 desaturase (omega-6 fatty acid desaturase), acyl-[acyl-carrier-protein] desaturase and acyl-CoA oxidase, implying the synthesis of unsaturated fatty acids (Additional file 2: Figure S4). The genes classified into fatty acid degradation by KEGG include long-chain acyl-CoA synthetase, acyl-CoA oxidase, alcohol dehydrogenase class-3, acetyl-CoA c-acetyltransferase, and glyoxysomal fatty acid beta-oxidation multifunctional protein MFP-a. Among them, long-chain acyl-CoA synthetase and acyl-CoA oxidase are also classified into fatty acid biosynthesis, but the up-regulation of the latter three genes implies the degradation of fatty acid.

**Table 4:** Fatty acid and lipid metabolism related terms enriched by KEGG Pathway

		samples		
		0.5 h	1 h	2 h
(Fatty acid)				
KEGG_ID	KEGG_term	Number of Genes involved	Number of Genes involved	Number of Genes involved
ko00061	Fatty acid biosynthesis	7^a^	7^a^	5
ko00071	Fatty acid degradation	1	2	7^a^
ko01040	Biosynthesis of unsaturated fatty acids	1	2	5
(Lipid)				
ko00564	Glycerophospholipid metabolism	1	8^a^	14^a^
ko00561	Glycerolipid metabolism	0	4	10^a^
(other)				
ko04144	endocytosis	3	8	24^a^

^a^indicates significantly enriched

On the other hand, the number of up-regulated genes involved in Glycerophospholipid and glycerolipid metabolism increase constantly with the increasing of stress time (Table 4). Genes catalyzing the formation of phospatidylcholine (lecithin), phosphatidylethanolamine, phosphatidyl-L-serine, phosphatidylglycerol, and phosphatidyl-D-myo-inositol are significantly up-regulated on 2-h of stress (Additional file 1: Table S4), indicating the synthesis of glycerophospholipid on 2-h of stress. Study reported that fatty acids of *Dunaliella salina* plasma membrane and microsomal undergo changes in desaturation pattern under salt stress. They hypothesized that unsaturated fatty acids could help to keep fluidity of membrane when confronting salt stress [28]. So it is possible that the new synthesized unsaturated fatty acids might be incorporated into the new glycerophospholipids.

Interestingly, endocytosis, which can absorb and start the degradation of cell membrane [29], is significantly enriched on 2-h of stress (Table 4). When confronting salt stress, *Dunaliella* cells begin to shrink to cause excessive cell membrane. It is possible that the excessive membrane is degraded into building blocks for synthesis of new glycerophospholipid with unsaturated fatty acids, or further degraded for fuel energy metabolism.

### Amino acids and protein metabolism

Several amino acid metabolism pathways are enriched by KEGG Pathway analysis on up-regulated genes (Table 5), including some high abundant amino acids such as glutamine, glutamic acid, serine, alanine, proline, aspartic acid and asparagine. These high abundant amino acids were reported to be synthesized during abiotic stress to act as compatible osmolytes, precursors for secondary metabolites, or storage forms of organic nitrogen [30]. Several amino acid biosynthesis pathways are also enriched especially on 2-h of stress, including phenylalanine, tyrosine and tryptophan biosynthesis, lysine biosynthesis, valine, leucine and isoleucine biosynthesis, and histidine metabolism (Table 5). These are low abundant amino acids and the synthesis of these amino acids is energetically costly and requires multiple reaction steps [30]. With the stress time increasing, the number of genes increased in all the pathways except arginine biosynthesis which remains stable, the increasing is very slowly or stable from 0.5-h of stress to 1-h of stress and sharply from 1-h of stress to 2-h of stress (Table 5). The sharp increase of gene numbers implies that the synthesis of these low abundant amino acids at a significantly higher rate on 2-h of stress. Ribosome biogenesis and transcription are also significantly enriched on 2-h of stress (Table 6). Genes involved in the two biological processes are highly expressed on 2-h of stress (Additional file 2: Figure S5 and S6). These together implies the synthesis of new protein on 2-h of stress at a significantly higher rate, compared with that of 0.5-h and 1-h of stress. Spliceosome is significantly enriched on 1-h and 2-h of stress (Table 6; Additional file 2: Figure S7). Post-transcriptional regulation mediated by spliceosome is an important way of gene regulation and was reported to involve in responses to various abiotic stress including salt and temperature stress [31, 32]. Alternative splicing is a fast way to generate new mRNAs (splice variants) using the existed pre-mRNAs, and then these splice variants can be translated into different proteins. Protein processing in endoplasmic reticulum is also significantly enriched on 1-h and 2-h of stress (Table 6; Additional file 2: Figure S8), which implies the regulation at post-translational level.

**Table 5:** Amino acid metabolism related terms enriched by KEGG Pathway

KO_Term	KO_ID	samples		
		0.5 h	1 h	2 h
		Number of Genes involved	Number of Genes involved	Number of Genes involved
Glycine, serine and threonine metabolism	ko00260	11^a^	15^a^	22^a^
Alanine, aspartate and glutamate metabolism	ko00250	9	11	17
Arginine and proline metabolism	ko00330	2	5	11^a^
Cysteine and methionine metabolism	ko00270	11^a^	13	24^a^
Phenylalanine, tyrosine and tryptophan biosynthesis	ko00400	4	4	14
Lysine biosynthesis	ko00300	1	4	9^a^
Valine, leucine and isoleucine biosynthesis	ko00290	0	2	12^a^
Histidine metabolism	ko00340	0	0	4
Arginine biosynthesis	ko00220	6	5	7

^a^indicates significantly enriched (*P* value ≤0.05)

**Table 6:** Protein metabolism related terms enriched by GO and KEGG Pathway

		samples		
		0.5 h	1 h	2 h
(transcription)				
GO_ID	GO_term	Number of Genes involved	Number of Genes involved	Number of Genes involved
GO:0006351	transcription, DNA-templated	26	56	191
GO:0006352	DNA-templated transcription, initiation	3	15	54^a^
GO:0006355	regulation of transcription, DNA-templated	22	42	125
(translation)				
GO:0006412	translation	50	51	127
GO:0006413	translational initiation	1	1	29
GO:0006417	regulation of translation	3	5	37
(other process)				
KO_ID	KO_term	Number of Genes involved	Number of Genes involved	Number of Genes involved
ko03008	Ribosome biogenesis in eukaryotes	0	1	33^a^
ko03040	Spliceosome	9	20^a^	74^a^
ko04141	Protein processing in endoplasmic reticulum	11	30^a^	51^a^
ko04120	Ubiquitin mediated proteolysis	1	2	13^a^

^a^indicates significantly enriched (*P* value ≤0.05)

On the other hand, ubiquitin mediated proteolysis is significantly enriched on 2-h of stress, which implies the degradation of proteins (Table 6; Additional file 2: Figure S9). The enrichments of protein synthesis and degradation at the same time implies a higher protein turnover rate on 2-h of stress. Some proteins are destined to degradation, such as misfolded proteins caused by stress [33], while new proteins are needed to adapt to the hypersaline environmental, such as chaperones (heat shock proteins) and reactive oxygen species scavenging enzymes (superoxide dismutase, catalase, and peroxiredoxins) [34]. Consistently, the expressions of these genes are up-regulated (Additional file 1: Table S4; Additional file 3).

### Ion homeostasis

Surprisedly, the expression of all the possible Na^+^ and Cl^−^ transporters or channels are not up-regulated up to 2-h of stress (Table 7), which implies that the enhancement of exclusion of Na^+^ or Cl^−^ are not needed, probably because the extracellular ions can’t go into *Dunaliella* cell easily. When confronting salt stress, *Dunaliella* cells undergo rapid shrinking by efflux of water through aquaporin [1]. The cell shrinkage and efflux of water result in the increased solutes concentrations in cytoplasm, and at the same time the glycerol synthesis starts, which contributes to the osmotic balance between cell membrane. From this point of view, if *Dunaliella* cells can restrict ion permission, the enhanced exclusion of the ions by membrane transporters seems no need, so we can’t see the up-regulation of these ion transporters.

**Table 7:** The expressions of the possible sodium and chloride ion transporters or channels identified in the transcriptiome

Gene ID	Swissprot Description	PFAM description	log2(FoldChange)^a^		
			0.5 h vs 0 h	1 h vs 0 h	2 h vs 0 h
(Sodium channels)					
i0_LQ_MIX7|c93492/f1p0/580	Sodium/sulfate cotransporter	–	FALSE	FALSE	FALSE
i2_LQ_MIX7|c2141/f1p8/2443	Sodium-dependent phosphate transporter	–	FALSE	FALSE	FALSE
i1_LQ_MIX7|c73474/f1p0/1231	sodium/metabolite cotransporter	–	FALSE	FALSE	FALSE
i1_LQ_MIX7|c78858/f1p0/1342	Probable sodium/sulfate cotransporter	–	FALSE	FALSE	FALSE
i1_HQ_MIX7|c19950/f2p0/1494	Sodium channel protein 60E	–	FALSE	FALSE	FALSE
i1_LQ_MIX7|c50163/f1p0/1376	sodium/metabolite cotransporter	–	FALSE	FALSE	FALSE
i1_LQ_MIX7|c105505/f1p0/1621	sodium/metabolite cotransporter	–	FALSE	FALSE	FALSE
i0_LQ_MIX7|c223101/f1p0/398	Sodium/calcium exchanger	–	FALSE	FALSE	FALSE
i2_LQ_MIX7|c1504/f1p0/2682	Urea-proton symporter	–	FALSE	DOWN	DOWN
i1_LQ_MIX7|c78130/f1p0/1314	–	Sodium/calcium exchanger protein	FALSE	FALSE	FALSE
i0_LQ_MIX7|c11594/f1p1/871	–	Sodium / potassium ATPase beta chain	FALSE	FALSE	FALSE
i0_LQ_MIX7|c172741/f1p0/649	–	Sodium/glutamate symporter//Ureide permease	FALSE	FALSE	FALSE
i0_LQ_MIX7|c4443/f1p1/741	–	Amiloride-sensitive sodium channel	FALSE	FALSE	FALSE
i1_LQ_MIX7|c29086/f1p0/1191	–	Sodium/calcium exchanger protein	FALSE	FALSE	FALSE
i1_LQ_MIX7|c27958/f1p0/1854	–	Sodium ion transport-associated//Rer1 family	FALSE	FALSE	FALSE
i0_LQ_MIX7|c5202/f1p0/598	–	Sodium:neurotransmitter symporter family	FALSE	FALSE	DOWN
i1_LQ_MIX7|c13817/f1p0/1659	–	Amiloride-sensitive sodium channel//EGF-like domain	FALSE	FALSE	FALSE
i0_HQ_MIX7|c12934/f2p1/896	–	Na+/H+ ion antiporter subunit	FALSE	FALSE	FALSE
(chloride channels)					
i1_LQ_MIX7|c29481/f1p0/1076	Chloride channel protein CLC-f	–	FALSE	FALSE	FALSE
i2_LQ_MIX7|c3324/f1p0/2034	Chloride channel protein CLC-f	–	FALSE	FALSE	FALSE
i1_LQ_MIX7|c13569/f1p0/1372	–	Dimerisation domain of Ca + −activated chloride-channel, anoctamin	FALSE	FALSE	FALSE

^a^“FALSE” means no significant up- or down-regulation, “DOWN” means significant down-regulation

Some other metal ion transporters are also not up-regulated on 0.5-h and 1-h of stress (Additional file 1: Table S5), while some metal ion transporters such as magnesium, zinc, and boron transporters are up-regulated on 2-h of stress. It is possible that the up-regulation of these transporters is to uptake metal ion for protein synthesis since *Dunaliella* cells began to synthesize large amounts of protein on 2-h of stress (Table 6).

### Salt stress sensing and signal transduction

Kinetic of glycerol synthesis in *Dunaliella* shows that the synthesis can be detected within minutes and is independent of protein synthesis [35], which indicates that the expression of the signal receptors and signal transducers might not be changed upon signal stimulation. Therefore, using the GO enrichments of the up-regulated genes to show the involved signaling pathways may not reflect the real truth. So far, very little has been known about the sensing and signal transduction cascades of salt stress in *Dunaliella*. For this reason, we tried to search the possible cell surface receptors based on the sequence annotation data. Two possible receptors are found: a putative G-protien coupled seven-transmembrane receptor (GPCR) and a receptor-like serine/threonine protein kinase. Small GTP-binding proteins were identified in *D. salina* plasma membrane by Mass Spectrometry and were thought to involve in sensing and signaling of salt stress in *D. salina* [8]. However, the ligands of the two receptors are less likely to be Na^+^ or Cl^−^. Due to the properties of salt stress, we focused on ion channels. Genes encoding sodium channel, chloride channel and mechanosensitive ion channel are found in the sequence data.

One gene is annotated as a sodium channel, which shows sequence similarity with Sodium channel protein 60E from *Drosophila melanogaster*. Sodium channel protein 60E is a voltage-gated sodium channel, and the opening and closing of which is triggered by changing the ion concentration, and hence the charge gradient, between the two sides of the cell membrane [36]. In rabbit chemoreceptor cells, the activation of voltage-gated sodium channels can cause cell membrane depolarization, which will result in Ca^2+^ influx by activation of voltage-dependent Ca^2+^ channels, so the signal is switched to intracellular Ca^2+^ signal [37, 38]. No sequences encoding voltage-dependent Ca^2+^ channel are found in the sequence data. It cannot be concluded that there are no voltage-dependent Ca^2+^ channels in *Dunaliella salina* since about 40% of the transcriptome cannot be annotated by the present database. Instead, sequences encoding sodium/calcium exchanger, Ca^2+^ transporting ATPase and calcium uniporter are found in the sequence data. Furthermore, sequences encoding Calcium dependent kinases, Ca^2+^/calmodulin-dependent protein phosphatase, Calcium sensing receptor, calmodulin, Calcium/calmodulin-dependent 3′, 5′-cyclic nucleotide phosphodiesterase are also found in the sequence data. These indicate that Ca^2+^ plays an important role in signal transduction.

Two genes are annotated as voltage gated chloride channel. Since the salt stress was applied by only increasing the concentration of NaCl in medium, we wonder if the chloride channel can sense the salt stress by the influx of Cl^−^. WNK kinase (serine/threonine-protein kinase) is reported to function as a direct chloride sensor, and several genes encoding WNK are also found in the transcriptome. WNK are activated by reduction in intracellular Cl^−^ concentrations and inactivated by binding of a Cl^−^. Chloride binding inhibited the autophosphorylation of WNK1, thereby inhibiting kinase activity [39, 40]. WNK is also reported to inhibit sodium [41, 42] and potassium channel [43, 44]. Except for these, how the influx of Cl^−^ triggers intracellular pathways are almost unknown.

Mechanosensitive ion channels will respond to the mechanical deformation, which includes changes in the tension, thickness, or curvature, of the membrane. Mechanosensitive channels respond to membrane tension by altering their conformation between an open state and a closed state [45, 46]. When confronted with salt stress, *Dunaliella* cells shrink rapidly, thus the membrane deformation could cause the opening of mechanosensitive ion channels. One gene showing significant sequence similarity with the mechanosensitive ion channel protein 5 of *Arabidopsis thaliana* is found; however we don’t know whether it is a calcium channel or other ion channel. The applying of salt stress can trigger the voltage-gated sodium channel, the voltage-gated chloride channel and also the mechanosensitive ion channel, it is hard to tell which one is the very first signal receptor. *Dunaliella* cells undergo rapid shrinkage by efflux of water through aquaporin [1]. So the mechanosensitive ion channel seems unlikely to be the first receptor, but might be the enhancer.

A previous paper showing that MAPK signaling cascade may be involved in the salt stress of *Dunaliella* by western blot analysis [47]. Many genes on MAPK signaling cascade are also found in the sequence data, including mitogen-activated protein kinase (MAPK), Mitogen-activated protein kinase kinase (MAP 2 K), Mitogen-activated protein kinase kinase kinase (MAP 3 K), Mitogen-activated protein kinase kinase kinase kinase (MAP 4 K), MAP kinase phosphatase 1, MAP kinase phosphatase 5, MAP kinase phosphatase 6, and dual specificity MAP kinase phosphatase, which indicates that MAPK signaling pathway may be involved in salt stress signal transduction of *Dunaliella salina*.

## Discussion

Most of the qPCR results shew up-regulations on at least one of the three time points of hypersaline stress, which is consistent with the NGS results except catalase and acetyl-CoA carboxylase. No up-regulations of catalase and acetyl-CoA carboxylase were detected by qPCR (Additional file 3). Acetyl-CoA carboxylase, which converts cytosol acetyl-CoA into malonyl CoA, the first committed step in the synthesis of fatty acids, is a multi-subunit enzyme in the chloroplasts of most plants and algae [48]. The up-regulation of acetyl-CoA carboxylase beta subunit was detected by qPCR. The up-regulation of its beta subunit could possibly imply the enhancement of the holoenzyme activity.

*Dunaliella* is a good model organism for studying salt tolerance. Omics methods are particularly appropriate to analyze the stress response of *Dunaliella* at a global level, because *Dunaliella* is a unicellular alga and there will be no problem caused by different cell types. Previous omics studies can’t give a global understanding of the response process of *Dunaliella* to salt stress due to limited discovery of genes involved or unappropriated time duration of stress [13, 14]. Here we reported the transcriptomic profiles on 0.5-h, 1-h and 2-h of hypersaline stress and compared the profiles with that before stress. Our results imply that the response of *Dunaliella* to hypersaline stress is almost finished on 2-h stress. The reasons are that nuclear origin of replication recognition complex and reproductive process are significantly enriched on 2-h stress, which implies that the algae cell starts to reproduce on 2-h of stress and cells already adapted the new environment. This is in agreement with the previous studies reporting that intracellular glycerol synthesis accomplished and cells recovered their original volume in about 2 h after hypersaline stress [15, 16].

When the hypersaline stress is applied, many of the genes classified into photosynthesis show a pulse expression pattern (quick increasing and then quick decreasing), implies that hypersaline stress induces enhancement of photosynthesis, but no constant enhancement of photosynthesis is observed. Carbon fixation from photosynthesis has its upper limit under a given condition. Study reported that only a small part of carbon source of glycerol synthesis comes from CO_2_ fixation of photosynthesis, and others are from starch breakdown [49]. However, constant increasing of carbon fixation is observed, this may be induced by NaHCO_3_ in the high salt medium when stress is applied. Constant increase of pigment biosynthetic process including heme biosynthetic process is also observed. Pigments such as carotenoids play a role in response to abiotic stress [50], and heme could be used to synthesize heme-containing enzymes, such as catalase and ascorbate peroxidase, which play important roles in detoxification of reactive oxygen species (ROS) [25]. Starch is the main carbon source of glycerol synthesis when hypersaline stress is applied [50]. Consistently, starch and sucrose metabolism is significantly enriched. The new drawn glycerol synthesis pathway includes only 6 enzyme-catalyzing steps to convert starch to glycerol, which is theoretically faster than previous reported pathways [9, 51]. Synthesis of unsaturated fatty acid is consistent with previous report [28]. Interestingly, endocytosis is significantly enriched, together with the enrichment of Glycerophospholipid metabolism. We speculate that endocytosis could cause the degradation of excessive plasma membrane and help the synthesis of new glycerophospholipid with unsaturated fatty acids.

From amino acids and protein metabolism, we found an indication of sharp increasing of protein synthesis rate on 2-h stress based on the significant enrichment of ribosome biogenesis and transcription. Enhancement of regulation on protein synthesis is also implied by the significant enrichment of spliceosome and protein processing in endoplasmic reticulum. While, the significant enrichment of ubiquitin mediated proteolysis implies the degradation of proteins. Taken together, accelerated protein turnover rate on 2-h of stress is implied.

It is very interesting that no sodium and chloride channels or transporters are up-regulated during the period of 2-h of stress. No up-regulation of Na^+^/H^+^ antiporter was detected by qPCR (Additional file 3). This is in consistent with the reports of Na^+^/H^+^ antiporter, which regards Na^+^/H^+^ antiporter as the main player to eliminate Na^+^ during salt stress [7, 52]. We can’t see the up-regulation of Na^+^/H^+^ antiporter, maybe the activity of Na^+^/H^+^ antiporter is regulated at protein level instead of mRNA level. However, if *Dunaliella* cell can restrict Na^+^ permission, the exclusion of the ions by membrane transporters seems no need.

The time setting of stress gives us good results. We can see the constant increasing of the number of up-regulated genes with the increasing of stress time. Many of the results of functional enrichment are consistent with the previous studies, such as the enhancement of photosynthesis and carbon fixation [9], the degradation of starch for glycerol synthesis [49], and the desaturation of membrane lipid [28] when hypersaline stress is applied.

We also have some new findings, such as the enhancement of heme biosynthetic process and endocytosis, the accelerated protein turnover rate, the enhancement of spliceosome and protein processing in endoplasmic reticulum in response to hypersaline stress. These new findings shed light on the mechanism of salt tolerance of photosynthetic plants and may help improving salt tolerance of crops by genetic manipulation.

Finally, we like to acknowledge the limitation of this study, that is, all the analyses are based on the transcriptomic data, the changes at mRNA level. Although protein levels are usually positively correlated with the mRNA levels, there are post-transcriptional and translational regulations that may affect the correlation. From this point of view, it is necessary to further figure out how a new intracellular balance at the physiological levels achieved in *Dunaliella salina* to adapt to hypersaline environment.

## Conclusion

At global transcriptomic level, the response of *Dunaliella salina* to hypersaline stress is systematic, possibly involving enhancement of photosynthesis, constant increasing of carbon fixation and heme biosynthetic process, degradation of starch, synthesis of glycerol, membrane lipid desaturation, accelerated protein turnover, enhancement of spliceosome, protein processing in endoplasmic reticulum, and endocytosis. The changes of these biological processes will help to understand the achieved new balance adapting to the hypersaline environment.

## Methods

### Algae and culture conditions

*D. salina* strain CCAP 19/30 was obtained from Mariela A. González and Thomas Pröschold. The algae grew in a controlled-environment chamber at 20 °C with 14 h lighting and 10 h darkness. The composition of the growth medium was 1 M NaCl, 5.0 mM NaNO_3_, 5.0 mM MgSO_4__7H_2_O, 0.1 mM NaH_2_PO_4__2H_2_O, 1.0 mM KCl, 10.0 mM NaHCO_3_, 0.3 mM CaCl_2__2H_2_O and a mixture of micronutrients [53]. Total 1200 mL algae in log phase (about 10^6^ cells/mL) were divided into 12 bottles, 100 mL per bottle. Three bottles were used for control; the other 9 bottles were used for hyper-salt stress treatment. For hyper-salt stress treatment, each of the 9 bottles was added directly with 100 mL of high salt medium (containing 4 M NaCl) to form a final NaCl concentration of 2.5 M and was further cultured in light at 20 °C. Algae cells under stress were harvested on time of 0.5-h, 1-h, and 2-h for total RNA extraction. The control algae cells were also harvested for total RNA extraction. In all, there are 4 time points (4 groups), 0 h, 0.5 h, 1 h and 2 h, each time point with three replicates. In total 12 RNA samples were prepared from 12 algae samples.

### RNA extraction

Total RNA was extracted with TRIzol (Invitrogen, USA) by following the user manual. RNA degradation and contamination was monitored on 1% agarose gels. RNA purity and integrity was checked by NanoPhotometer (IMPLEN, Germany) and RNA Nano 6000 Assay Kit of the Bioanalyzer 2100 system (Agilent Technologies, USA). RNA concentration was measured by Qubit® 2.0 Flurometer together with Qubit® RNA Assay Kit (Life Technologies, USA).

### Hiseq libraries construction for the second generation sequencing

The 12 RNA samples were used to construct 12 Hiseq libraries, one library per RNA sample. Three microgram total RNA per RNA sample was used to prepare the sequencing library with NEBNext Ultra RNA Library Prep Kit for Illumina (NEB, USA) by following the manufacturer’s instruction. Briefly, mRNA was purified by using poly-T magnetic beads and was fragmented to perform first-strand cDNA by using M-MuLV reverse transcriptase and random hexamer-primers. Second-strand cDNA was synthesized by using DNA polymerase I and RNase H. The cDNA was end-repaired and A-tailed, and NEBNext Adaptor with hairpin loop structure was linked. After size selection with AMPure XP system (Beckman Coulter, Beverly, USA) and PCR amplification with Phusion High-Fidelity DNA polymerase, samples were sequenced on the Illumina Hi-Seq 2000 system, generating paired-end (PE) reads with a length of 150 bp.

### PacBio library construction for the third generation sequencing

The only reported *Dunaliella salina* (CCAP19/18) genome shows low sequence similarity with our algae strain (CCAP 19/30) and can’t be used as the reference genome for mRNA sequence assembly and annotation, so the third generation sequencing was applied to generate the full length mRNA sequences. One microgram mRNA (generated by equally mixing mRNAs from the 9 stress samples and the 3 control samples) were used for first-strand cDNA synthesis with SMARTer PCR cDNA Synthesis Kit (Clontech Cat. 634,926). Double-strand cDNA was amplified with PrimeSTAR GXL DNA Polymerase (Clontech R050B). The > 4 kb cDNA fraction was generated with the BluePippin Size Selection System and mixed with the cDNA before fractionation at 1:1 ratio. The mixed cDNA was used to construct a combined SMRTbell Libray for sequencing. The library was subsequently sequenced on the PacBio Sequel System platform using SMRT Cell 1 M v2.

### Data processing and transcriptome assembly

Firstly, circular consensus sequences (CCS) were generated from Pacbio raw reads. Then, full length non chimera (FLNC) reads were generated from CCS. FLNC reads should have sequence regarding to both 5′ and 3′ Clontech kit primers as well as a poly-A tail signal preceding the 3′ primer. FLNC reads were clustered by ICE (Iterative isoform-clustering) and a consensus sequence was generated from each cluster. The consensus sequences were corrected by non-full length sequences to generate the polished consensus sequences.

Hiseq raw reads were processed to generate Hiseq clean reads by removing adapter sequences, excluding reads contains > 50% nucleotides with Qphred ≤20, and reads with ambiguous bases N. Hiseq clean reads were used to correct the polished consensus sequences obtained from the third generation sequencing. The correction process was done by Proovread [54]. The final corrected unique full length sequences were used for annotation and gene expression analysis.

### Coding sequence analysis and gene annotation

Transcripts were firstly submitted to Nr and Swissprot protein database to analyze the potential open reading frames (ORF), then the transcripts failed to hit any proteins in the two databases were submitted to the software ESTSCAN (3.0.3) [55] to analyze the potential ORF, finally the transcripts failed the ESTSCAN are defined as non-coding sequences. The corrected unique full length sequences were annotated against 7 databases, Nr (NCBI’s non-redundant protein sequences), Nt (NCBI’s nucleotide sequences), Pfam (protein family), KOG/COG (clusters of orthologous groups of proteins/eukaryotic ortholog groups), Swiss-Prot, KEGG (Kyoto encyclopedia of genes and genomes) and GO (gene ontology) database. The softwares used are diamond v0.8.22 for Nr (e-value = 1e-5), KOG/COG (1e-3) and Swiss-Prot (e-value = 1e-5); NCBI blast 2.2.28 for Nt (e-value = 1e-5); HMMER 3.0 for Pfam (e-value = 0.01); KEGG automatic annotation server (KAAS) for KEGG (e-value = 1e-10), Blast2GO v2.5 for GO (e-value = 1e-6). When available, the functional annotation from Swiss-Prot was preferred because of its high accuracy.

### Quantification of gene expression level

Briefly, Hiseq clean reads from each replicate were mapped to the corrected unique full-length sequences using HTSeq v0.6.1 [56]. And then FPKM (expected number of Fragments per Kilobase of transcript sequence per Millions base pairs sequenced) of each gene was calculated based on the length of the gene and reads count mapped to this gene. FPKM considers the effect of sequencing depth and gene length at the same time, and is currently the most commonly used method for estimating gene expression levels [57]. Clustering analysis of gene expression value was performed by SOM method [58].

### Differential expression assessment

Differential expression analysis of two groups was performed by using the DESeq R package with the read counts from HTSeq [59]. DESeq provides statistical routines for determining differential expression in digital gene expression data, using a model based on the negative binomial distribution. The resulting *P* values were adjusted using the Benjamini and Hochberg’s approach for controlling the false discovery rate. Genes with an adjusted *P*-value < 0.05 found by DESeq were assigned as differentially expressed.

### Gene enrichment analysis

Gene Ontology (GO) terms were assigned to each genes based on the BLAST2GO [60]. Gene Ontology (GO) enrichment analysis of differentially expressed genes were implemented by the GOseq R package [61], in which gene length bias was corrected. GO terms with corrected P value < 0.05 were considered significantly enriched by differential expressed genes.

KEGG pathways were assigned to each genes based on the KAAS. KOBAS software [62] was used to test the statistical enrichment of differential expression genes in KEGG pathways. The enrichment analysis was tested using a hypergeometric test at a significance cutoff of 0.05 false discovery rate (FDR). Pathways were assigned as significantly enriched if they had a FDR below 5%.

### Real-time PCR (qPCR)

In order to confirm the expression results from RNA-sequencing, we perform qPCR analysis of the key genes from each functional group. The qPCR kit was from TaKaRa (DRR081A, TaKaRa Biotechnology, and Dalian, China). The primers used in qPCR were in the supplementary materials (Additional file 3). The reference gene used in qPCR was 18S rRNA. The relative expression quantification was calculated by the 2^–∆∆Ct^ method.

## Supplementary information

**Additional file 1: Table S1.** The data qualities of the libraries of the second-generation sequencing. **Table S2.** Transcripts properties from the third-generation sequencing. **Table S3.** GO terms enriched from the down-regulated genes. **Table S4.** Up-regulation of some key genes on different stress time compare with that before stress. **Table S5.** The expressions of the possible metal ion (excluding sodium and chloride ion) transporters or channels identified in the transcriptome.

**Additional file 2: Figure S1.** Clustering analysis of the differentially expressed genes in each sample. **Figure S2.** Heat-map of carbon fixation. **Figure S3.** heat-map of tetrapyrrole biosynthetic process. **Figure S4.** Heat-map of fatty acid biosynthesis. **Figure S5.** Heat-map of ribosome biogenesis. **Figure S6.** Heat-map of transcription. **Figure S7.** Heat-map of spliceosome. **Figure S8.** Heat-map of protein processing in endoplasmic reticulum. **Figure S9.** Heat-map of ubiquitin mediated proteolysis.

**Additional file 3.** Confirmation of the expressions of the key genes by qPCR.

## Data Availability

The datasets generated during the current study are deposited in the Sequence Read Archive under accession number SRR8552788, and SRR8543799 to SRR8543810. [https://trace.ncbi.nlm.nih.gov/Traces/sra/sra.cgi] The datasets will be available after the publishing of this manuscript or from the corresponding author on reasonable request.

## Abbreviations

ALDO: Fructose-bisphosphate aldolase
CCAP: The Culture Collection of Algae and Protozoa
CCS: Circular consensus sequences
CEGs: Core eukaryotic genes
CGT: Cyclomaltodextrin glucanotransferase
DHAP: Dihydroxyacetone phosphate
DsGPDH: *Dunaliella salina* glycerol-3-phosphate dehydrogenase
EST: Expressed sequence tag
FDR: False discovery rate
FLNC: Full length non chimera
FPKM: Expected number of Fragments per Kilobase of transcript sequence per Millions base pairs sequenced
GADP: Glyceraldehyde 3-phosphate
GO: Gene ontology
GPCR: G-protien coupled seven-transmembrane receptor
GPDH: Glycerol-3-phosphate dehydrogenase
GPI: Glucose-6-phosphate isomerase
ICE: Iterative isoform-clustering
ISA: Isoamylase
KAAS: KEGG automatic annotation server
KEGG: Kyoto encyclopedia of genes and genomes
KOG/COG: Clusters of orthologous groups of proteins/eukaryotic ortholog groups
MAP: Mitogen-activated protein
MAPK: Mitogen-activated protein kinase
Nr: NCBI’s non-redundant protein sequences
Nt: NCBI’s nucleotide sequences
ORF: Open reading frames
OtsA: Trehalose 6-phosphate synthase
OtsB: Trehalose 6-phosphate phosphatase
Pfam: Protein family
PGM: Phosphoglucomutase
PYG: Starch phosphorylase
ROS: Reactive oxygen species
TPI: Triosephosphate isomerase
WNK kinase: With no lysine (K) kinase
