# Supplementary material for: Transcriptomic profiles of Dunaliella salina in response to hypersaline stress
Source: BMC Genomics. 2020 Feb 3;21:115. doi: 10.1186/s12864-020-6507-2 (PMC6998148; doi:10.1186/s12864-020-6507-2)
Supplement: Supplementary file 1 — Additional file 1: Table S1. The data qualities of the libraries of the second-generation sequencing. Table S2. Transcripts properties from the third-generation sequencing. Table S3. GO terms enriched from the down-regulated genes. Table S4. Up-regulation of some key genes on different stress time compare with that before stress. Table S5. The expressions of the possible metal ion (excluding sodium and chloride ion) transporters or channels identified in the transcriptome. [file 12864_2020_6507_MOESM1_ESM.pdf]

Table S1, the data qualities of the libraries of the second-generation sequencing

Sample*	Raw Reads	Clean reads	Clean bases	Error (%)	Q20 (%)	Q30 (%)	GC (%)
0h_R1	64161390	61893000	9.28G	0.02	97.21	92.88	56.38
0h_R2	63970244	61949592	9.29G	0.01	97.28	93.03	56.53
0h_R3	71457828	68976474	10.35G	0.01	97.40	93.27	56.56
0.5h_R1	68365392	66752244	10.01G	0.01	97.98	94.70	56.45
0.5h _R2	49585896	49411914	7.41G	0.01	97.91	94.43	56.63
0.5h _R3	70751998	68453128	10.27G	0.01	97.94	94.62	56.62
1h_R1	62322764	60612712	9.09G	0.01	97.82	94.39	56.73
1h_R2	62349100	60604888	9.09G	0.01	97.96	94.64	56.79
1h_R3	63123074	61320462	9.2G	0.01	97.93	94.58	56.63
2h_R1	64791654	63006452	9.45G	0.01	97.97	94.67	56.67
2h_R2	60709782	58681302	8.8G	0.01	98.00	94.74	56.72
2h_R3	52398294	52216548	7.83G	0.01	97.86	94.33	56.40

*0h_R1, 0h_R2, and 0h_R3 represent three replicates of the 0-hour stress (no salt stress applied) group; 0.5h_R1, 0.5h_R2, and 0.5h_R3 represent three replicates of the 0.5-hour stress group; 1h_R1, 1h_R2, and 1h_R3 represent three replicates of the 1-hour stress group; 2h_R1, 2h_R2, and 2h_R3 represent three replicates of the 2-hour stress group;

Table S2, transcripts properties from the third-generation sequencing

	Transcripts length distribution				
	200-500 bp	500-1 kbp	1-2 kbp	>2 kbp	Total
Number of transcripts	6495	17950	17553	1866	43864
**Transcripts length statistics**					
Min Length (bp)	Mean Length (bp)	Median Length (bp)	Max Length (bp)	N50 (bp)	N90 (bp)
242	1009	918	8978	1175	587

Table S3, GO terms enriched from the down-regulated genes

GO_accession	Description	Number of Genes involved
0.5 h VS 0 h		
GO:0030246	carbohydrate binding*	8
GO:0005515	protein binding*	69
1 h VS 0 h		
GO:0006259	DNA metabolic process	44
GO:0005515	protein binding	103
GO:0005856	cytoskeleton	19
GO:0009101	glycoprotein biosynthetic process	12
GO:0006024	glycosaminoglycan biosynthetic process	11
GO:0030286	dynein complex	11
2 h VS 0 h		
GO:0005515	protein binding	272
GO:0016740	transferase activity	274
GO:0036211	protein modification process	90
GO:0006259	DNA metabolic process	104
GO:2001141	regulation of RNA biosynthetic process	117
GO:0005856	cytoskeleton	52
GO:0006024	glycosaminoglycan biosynthetic process	20
GO:0009101	glycoprotein biosynthetic process	29
GO:0010167	response to nitrate	26
GO:0015698	inorganic anion transport	32
GO:0006869	lipid transport	15
GO:0015074	DNA integration	18
GO:0006914	autophagy	21
GO:0005096	GTPase activator activity	13
GO:0030286	dynein complex	27

*not significantly enriched

Table S4, up-regulation of some key genes on different stress time compare with that before stress

Gene name	$\log_2$(FoldChange)*		
Swissprot Description	0.5h VS 0h	1h VS 0h	2h VS 0h
Carbonic anhydrase	0.53	0.41	0.53
Ribisco activase	FALSE	1.02	1.48
Catalase isozyme B	FALSE	FALSE	0.70
L-ascorbate peroxidase 8	FALSE	0.44	1.14
starch phosphorylase (PYG)	0.57	1.00	2.03
Phosphoglucomutase (PGM)	0.71	2.08	3.71
Glucose-6-phosphate isomerase (GPI)	FALSE	1.25	1.97
ATP-dependent 6-phosphofructokinase 5 (PFK1)	FALSE	FALSE	0.56
Triosephosphate isomerase (TPI)	0.66	1.04	1.43
Pyruvate kinase (PK)	FALSE	2.25	2.04
glycerol-3-phosphate dehydrogenase (DsGPDH2)	0.51	0.87	1.05
Acetyl-CoA carboxylase	0.52	0.55	FALSE
Delta-12 desaturase	FALSE	0.56	0.58
Acyl-ACP desaturase	0.58	0.63	0.14
Phosphatidyl-N-methylethanolamine N-methyltransferase	FALSE	FALSE	1.20
CDP-Ethanolamine:DAG ethanolamine phosphotransferase	FALSE	FALSE	3.53
Lysophospholipid acyltransferase	FALSE	FALSE	1.37
Heat shock 70 kDa protein (HSP70)	0.43	2.00	3.70
Heat shock protein 90 (HSP90)	FALSE	1.00	1.51
Heat shock protein 83 (Hsp83)	FALSE	0.62	0.53
Fructose-bisphosphate aldolase	FALSE	FALSE	0.80
Superoxide dismutase [Mn]	FALSE	0.66	1.06
Superoxide dismutase [Fe]	FALSE	0.56	1.22
Peroxiredoxin	FALSE	0.64	0.83
Beta-fructofuranosidase	FALSE	1.42	0.97
Na^+/H^+ antiporter	FALSE	FALSE	FALSE

*"FALSE" indicates not significantly up-regulated or down regulated.

Table S5, the expressions of the possible metal ion (excluding sodium and chloride ion) transporters or channels identified in the transcriptome

Gene ID	NR Description	Swissprot Description	log2(FoldChange)*		
			0.5h vs 0h	1h vs 0h	2h vs 0h
c108091/f1p0/1535	CDF transporter	--	FALSE	FALSE	FALSE
c7863/f1p0/1087	--	Copper transporter 5	FALSE	FALSE	FALSE
c2215/f6p0/1401	--	Fe(2+)/Mn(2+) transporter pcl1	FALSE	FALSE	FALSE
c55681/f3p0/1252	--	Magnesium transporter MgtE	FALSE	FALSE	UP
c202479/f1p0/1244	--	Magnesium transporter MRS2-1	FALSE	FALSE	FALSE
c5554/f1p0/1870	--	Magnesium transporter MRS2-A	FALSE	FALSE	UP
c157998/f1p0/1424	--	Molybdate-anion transporter	FALSE	FALSE	FALSE
c155710/f1p0/1468	--	Probable boron transporter 2	FALSE	FALSE	UP
c11961/f1p0/1648	--	Probable magnesium transporter NIPA5	FALSE	FALSE	FALSE
c73336/f1p0/1689	--	Probable metal-nicotianamine transporter	FALSE	FALSE	FALSE
c271360/f1p0/1019	--	Putative magnesium transporter MRS2-D	FALSE	FALSE	FALSE
c21210/f1p0/1462	--	Uncharacterized transporter C11D3.06	FALSE	FALSE	UP
c235505/f1p1/409	--	Vacuolar iron transporter homolog 1	FALSE	NA	NA
c47111/f1p0/1787	--	Zinc transporter 4	FALSE	FALSE	FALSE
c2107/f1p0/2339	--	Zinc transporter 7	FALSE	FALSE	UP
c20166/f2p0/1213	--	Zinc transporter ZTP29	FALSE	FALSE	DOWN
c157724/f1p0/1157	--	Zinc transporter ZupT	FALSE	FALSE	FALSE
c2575/f1p0/2693	--	ABC transporter B family member 28	FALSE	FALSE	UP
c118548/f2p0/1585	--	ABC transporter C family member 11	FALSE	FALSE	UP
c78031/f1p0/1808	--	ABC transporter E family member 2	FALSE	FALSE	FALSE
c202919/f1p0/1467	--	ABC transporter F family member 2	FALSE	FALSE	DOWN
c89604/f1p0/962	--	ABC transporter F family member 3	FALSE	FALSE	FALSE
c244316/f1p0/969	--	ABC transporter F family member 4	FALSE	FALSE	UP
c116462/f1p0/1155	--	ABC transporter G family member 21	FALSE	FALSE	FALSE
c158998/f1p0/1778	--	ABC transporter G family member 22	DOWN	FALSE	UP
c2842/f1p0/2112	--	ABC transporter G family member 9	DOWN	FALSE	UP

c3652/f1p1/759	--	ABC transporter I family member 10	FALSE	FALSE	FALSE
c106076/f1p0/1615	--	ABC transporter I family member 11	FALSE	FALSE	FALSE
c154944/f1p0/1507	--	ABC transporter I family member 20	DOWN	DOWN	DOWN
c51535/f1p0/1387	--	ABC transporter I family member 6	FALSE	FALSE	FALSE
c379117/f1p0/783	--	Ion channel CASTOR	FALSE	FALSE	FALSE
c117188/f1p0/1573	--	Mechanosensitive ion channel protein 5	FALSE	FALSE	UP

*”FALSE” means no significant up- or down-regulation, “UP” means significant up-regulation, “DOWN" means significant down-regulation, and “NA” means not available.
