# Supplementary material for: Transcriptomic profiles of Dunaliella salina in response to hypersaline stress
Source: BMC Genomics. 2020 Feb 3;21:115. doi: 10.1186/s12864-020-6507-2 (PMC6998148; doi:10.1186/s12864-020-6507-2)
Supplement: Supplementary file 2 — Additional file 2: Figure S1. Clustering analysis of the differentially expressed genes in each sample. Figure S2. Heat-map of carbon fixation. Figure S3. heat-map of tetrapyrrole biosynthetic process. Figure S4. Heat-map of fatty acid biosynthesis. Figure S5. Heat-map of ribosome biogenesis. Figure S6. Heat-map of transcription. Figure S7. Heat-map of spliceosome. Figure S8. Heat-map of protein processing in endoplasmic reticulum. Figure S9. Heat-map of ubiquitin mediated proteolysis. [file 12864_2020_6507_MOESM2_ESM.pdf]

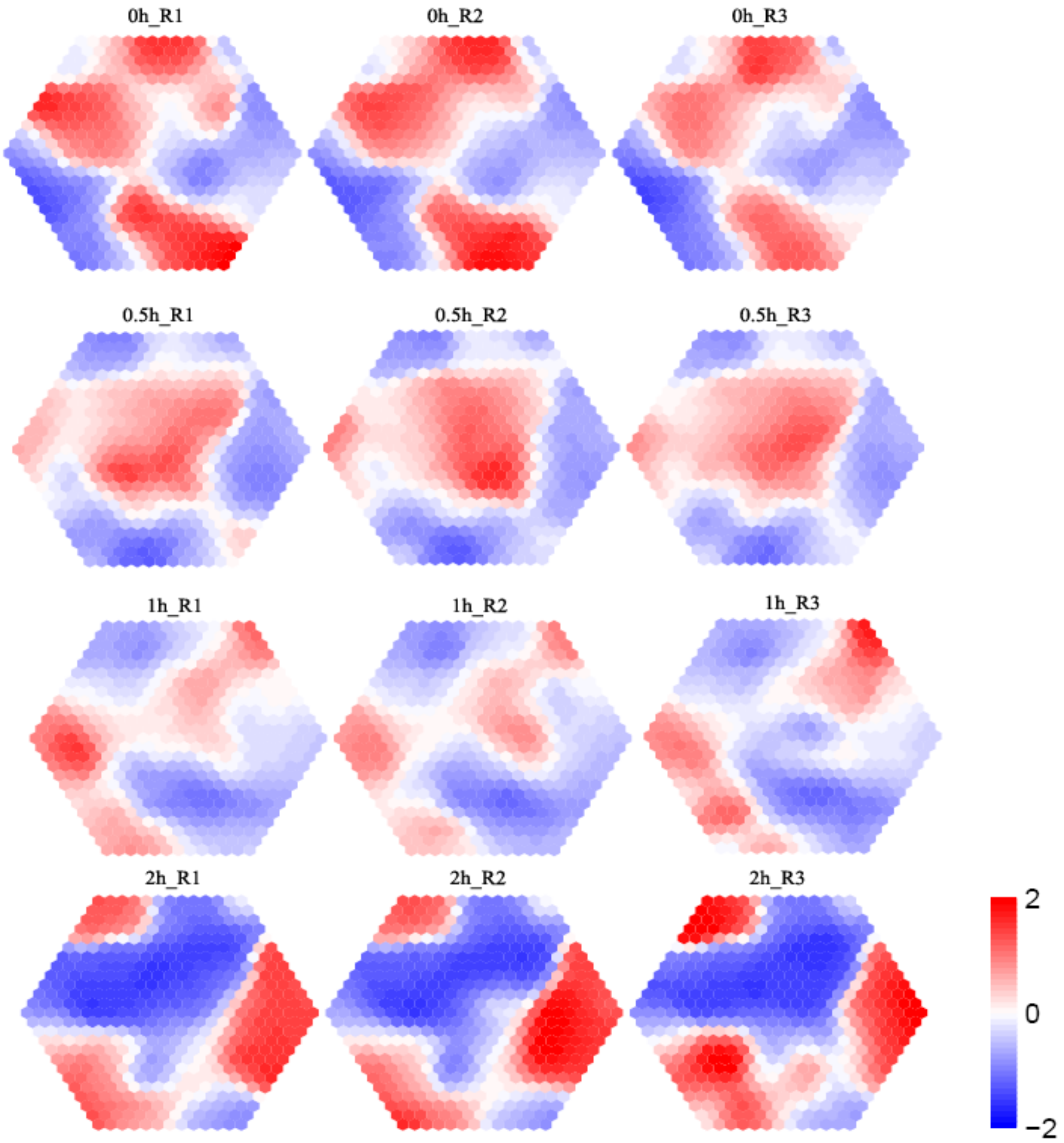

Figure S1, clustering analysis of the differentially expressed genes in each sample, all the differentially expressed genes are used for clustering analysis based on their expression values generated from FPKM. The red represents high expression value and the blue represents low expression value. Within each sample (the big hexagon), genes with the same or similar expression patterns are mapped to the same or nearby map nodes (the small hexagon). 0h_R1, 0h_R2, and 0h_R3 represent three replicates of the 0-hour stress (no salt stress applied) group; 0.5h_R1, 0.5h_R2, and 0.5h_R3 represent three replicates of the 0.5-hour stress group; 1h_R1, 1h_R2, and 1h_R3 represent three replicates of the 1-hour stress group; 2h_R1, 2h_R2, and 2h_R3 represent three replicates of the 2-hour stress group; It is clear that replicates within the same group have similar expression profiles.

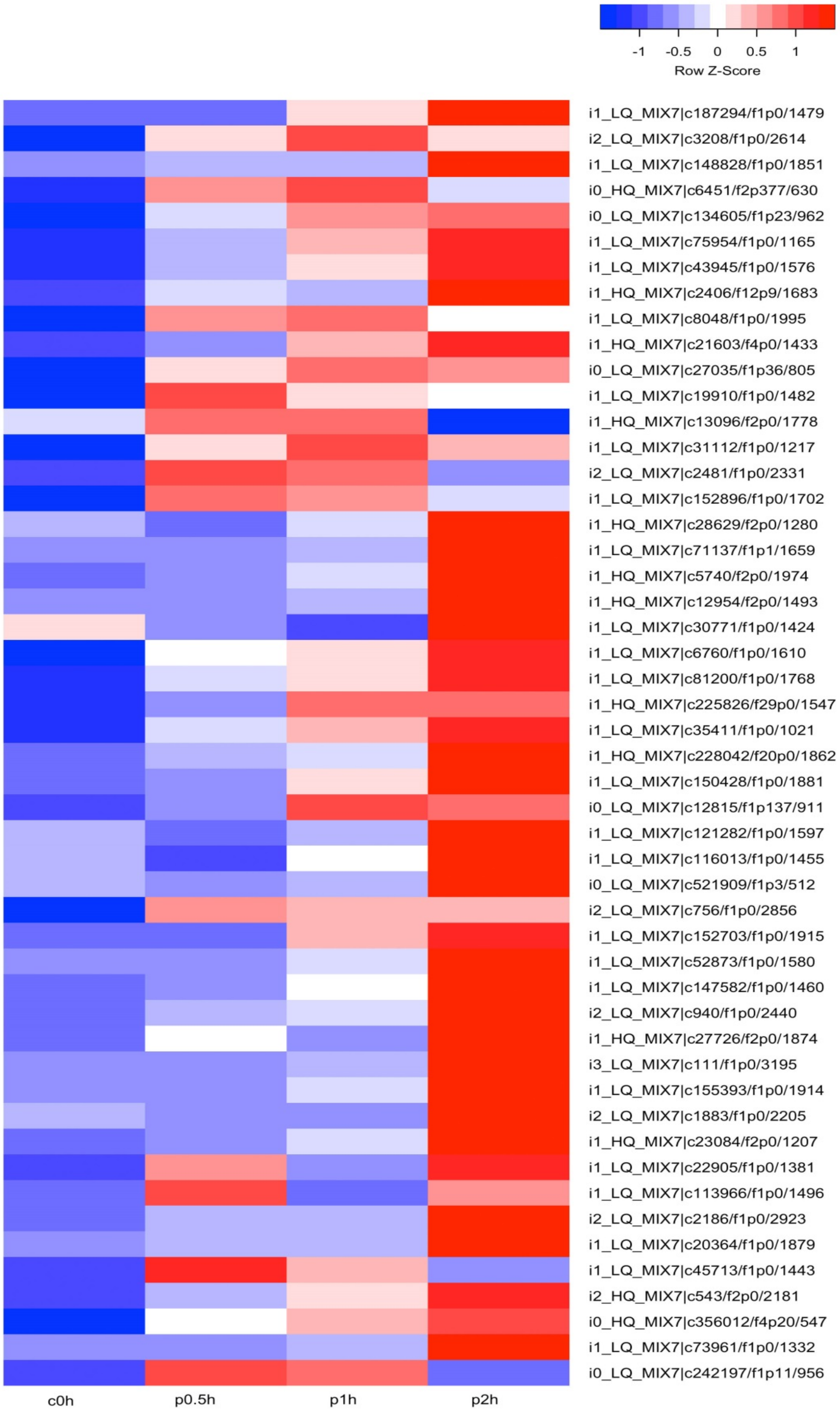

Figure S2, heat-map of carbon fixation. the colors from blue to red represent the gene express values from low to high. The z-scores represent gene expression values were generated from their FPKMs. The four columns represent the four experimental groups. C0h represents the control group with no hypersaline stress applied. p0.5h, p1h, and p2h represent the three hypersaline treated groups with 0.5-hour, 1-hour, and 2-hour time duration. Genes IDs are on the right. Genes are also grouped base on their expression patterns.

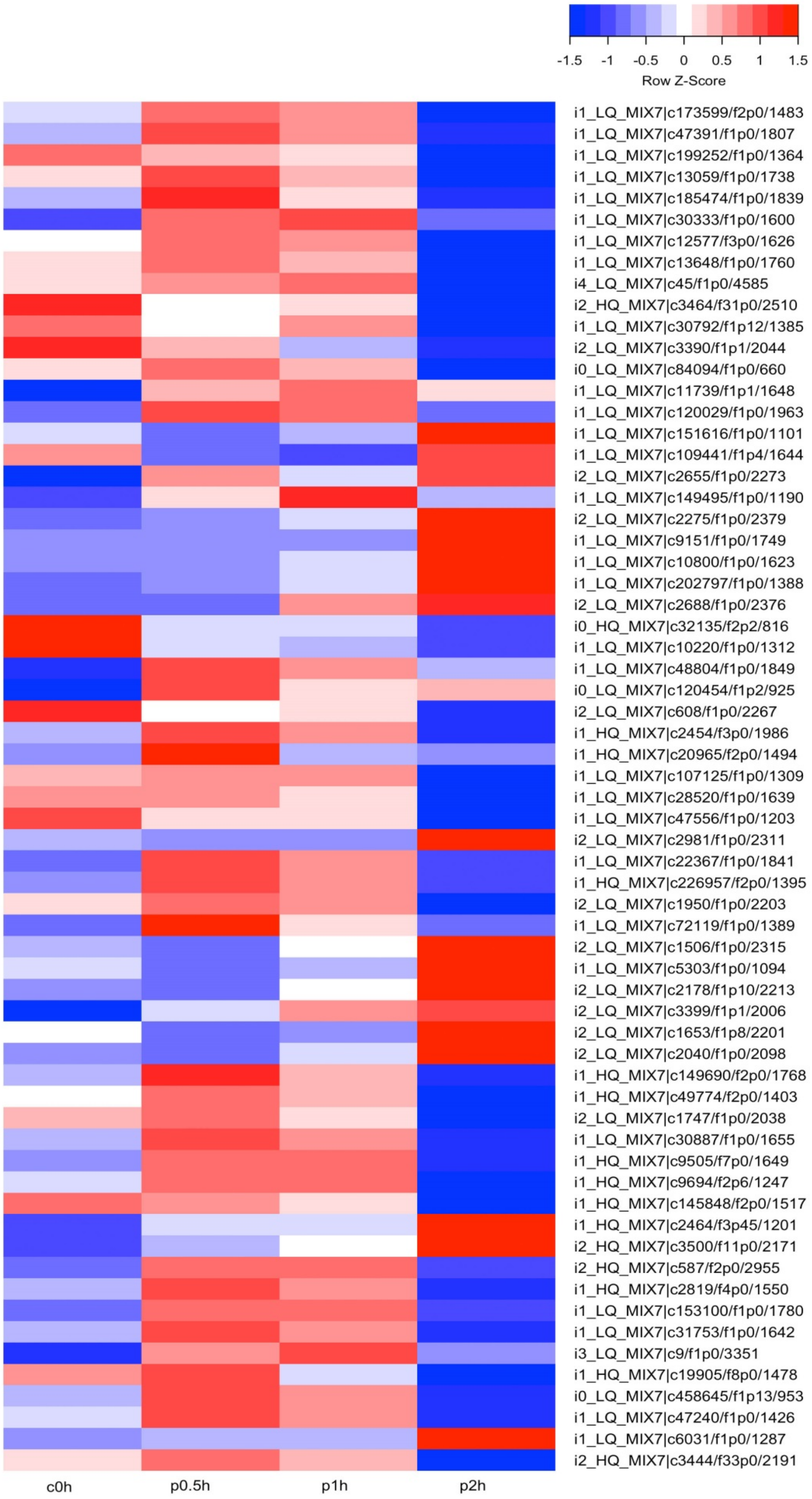

Figure S3, heat-map of tetrapyrrole biosynthetic process. Please refer to Fig. S2 for figure legends.

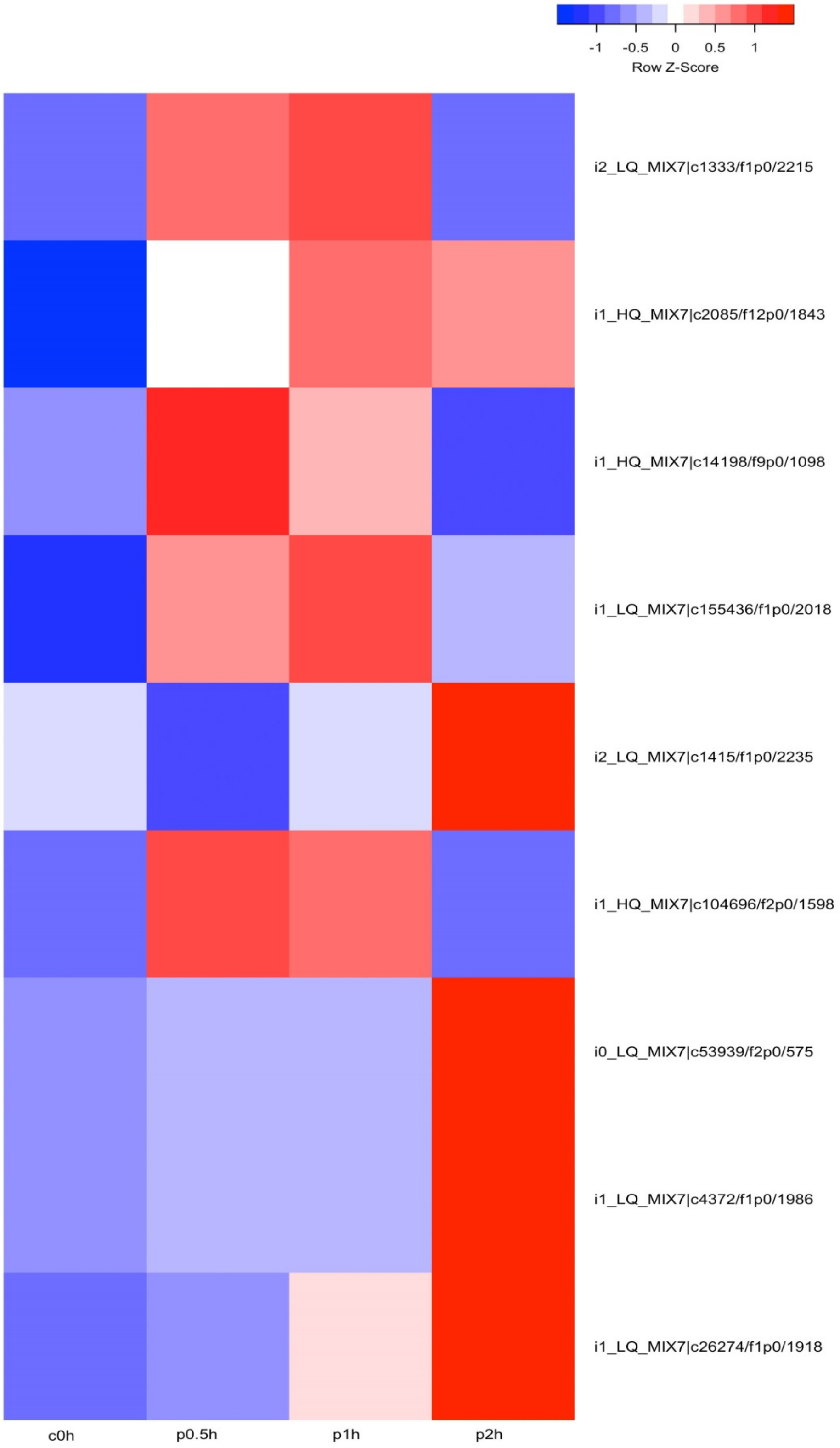

Figure S4, heat-map of fatty acid biosynthesis. Please refer to Fig. S2 for figure legends.

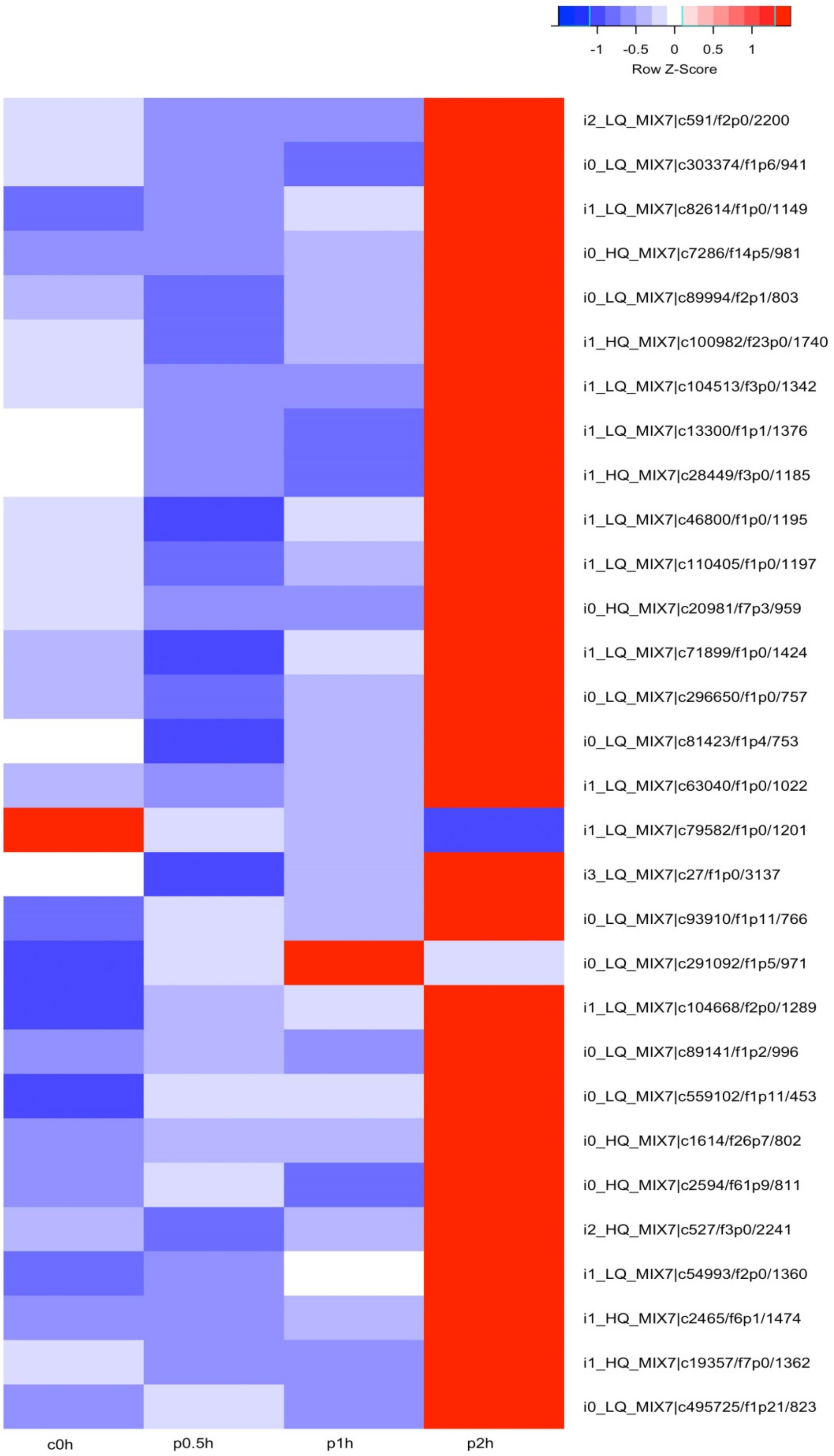

Figure S5, heat-map of ribosome biogenesis. Please refer to Fig. S2 for figure legends.

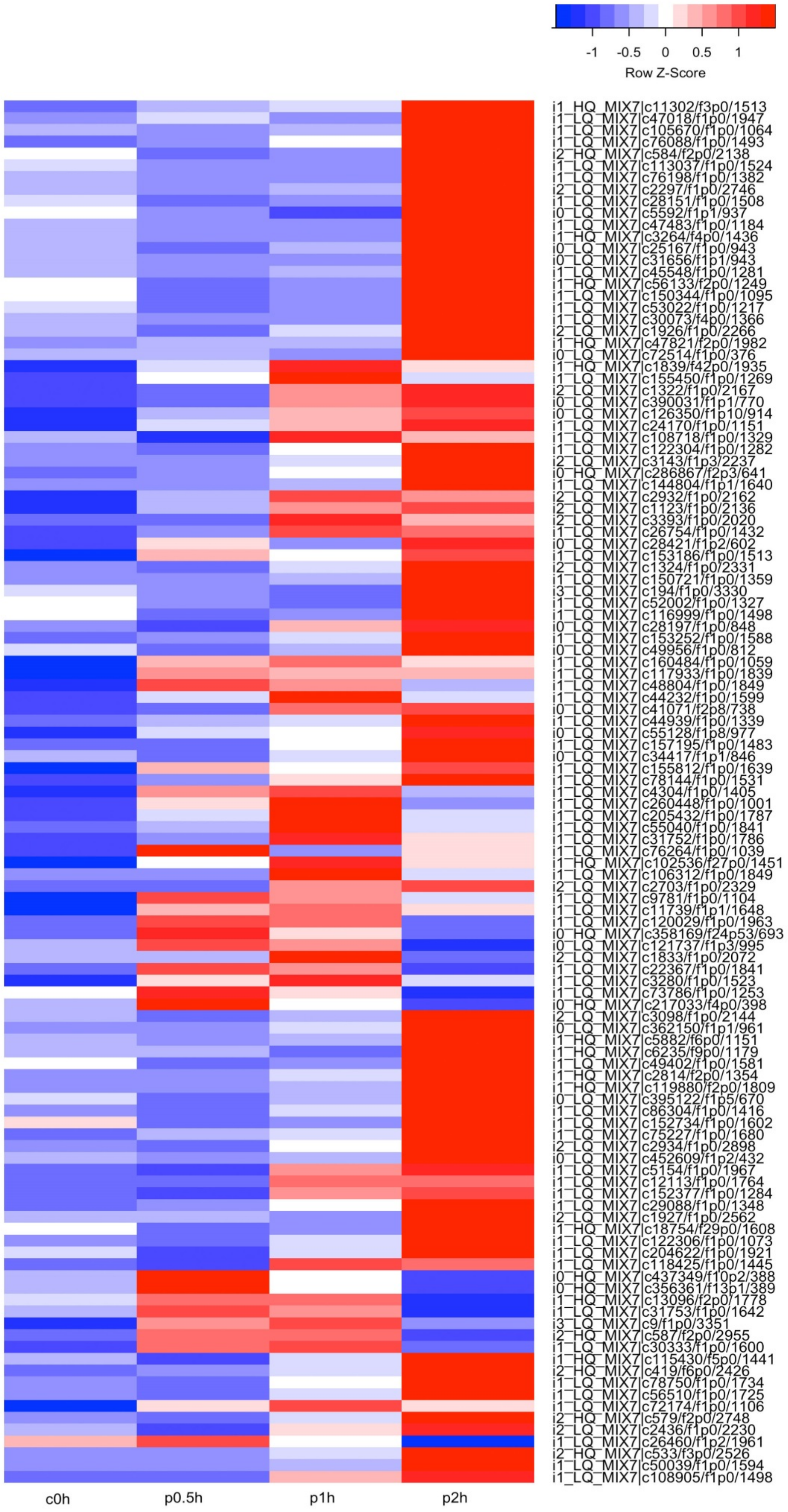

Figure S6, heat-map of transcription. Please refer to Fig. S2 for figure legends.

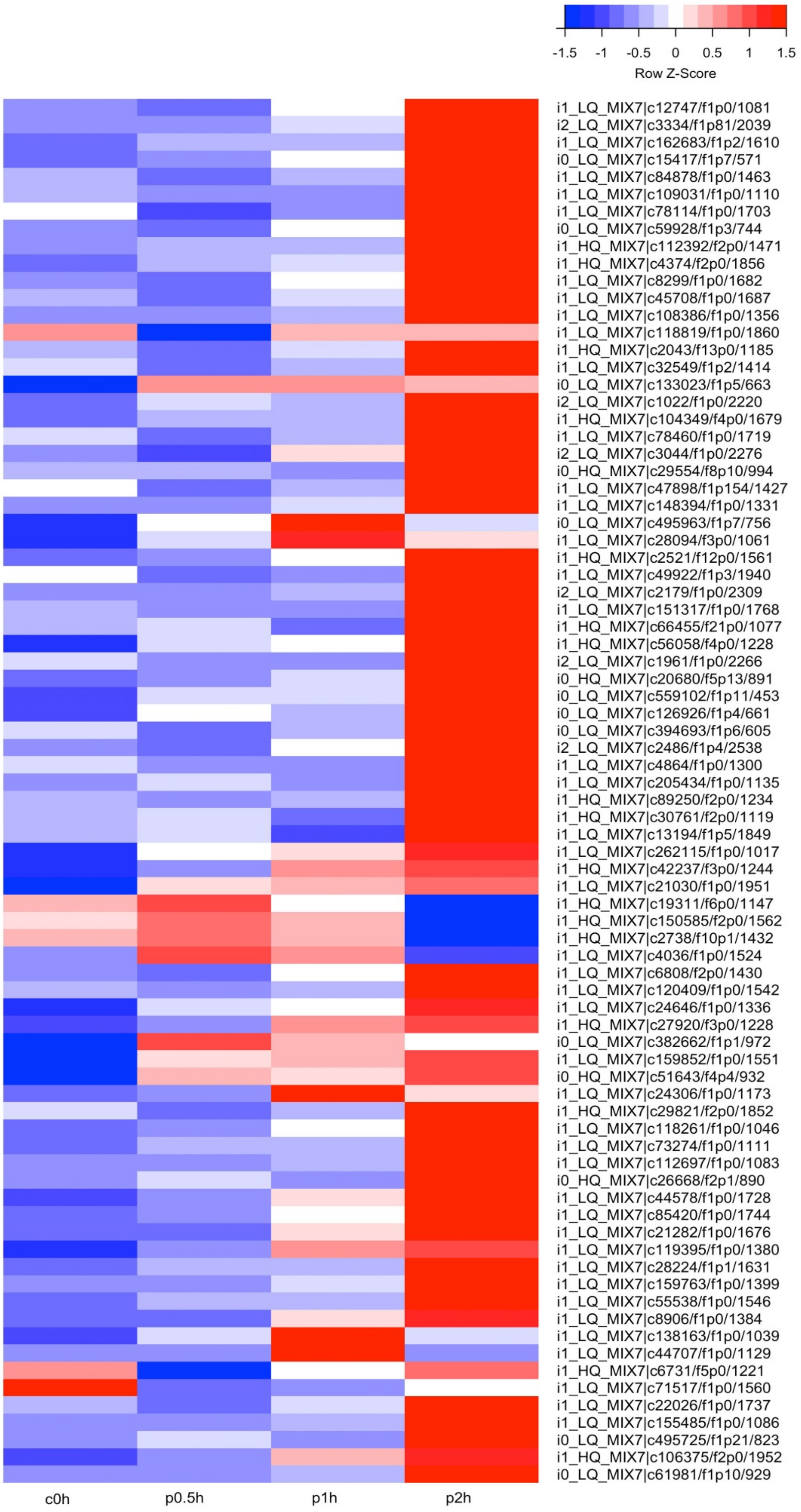

Figure S7, heat-map of spliceosome. Please refer to Fig. S2 for figure legends.

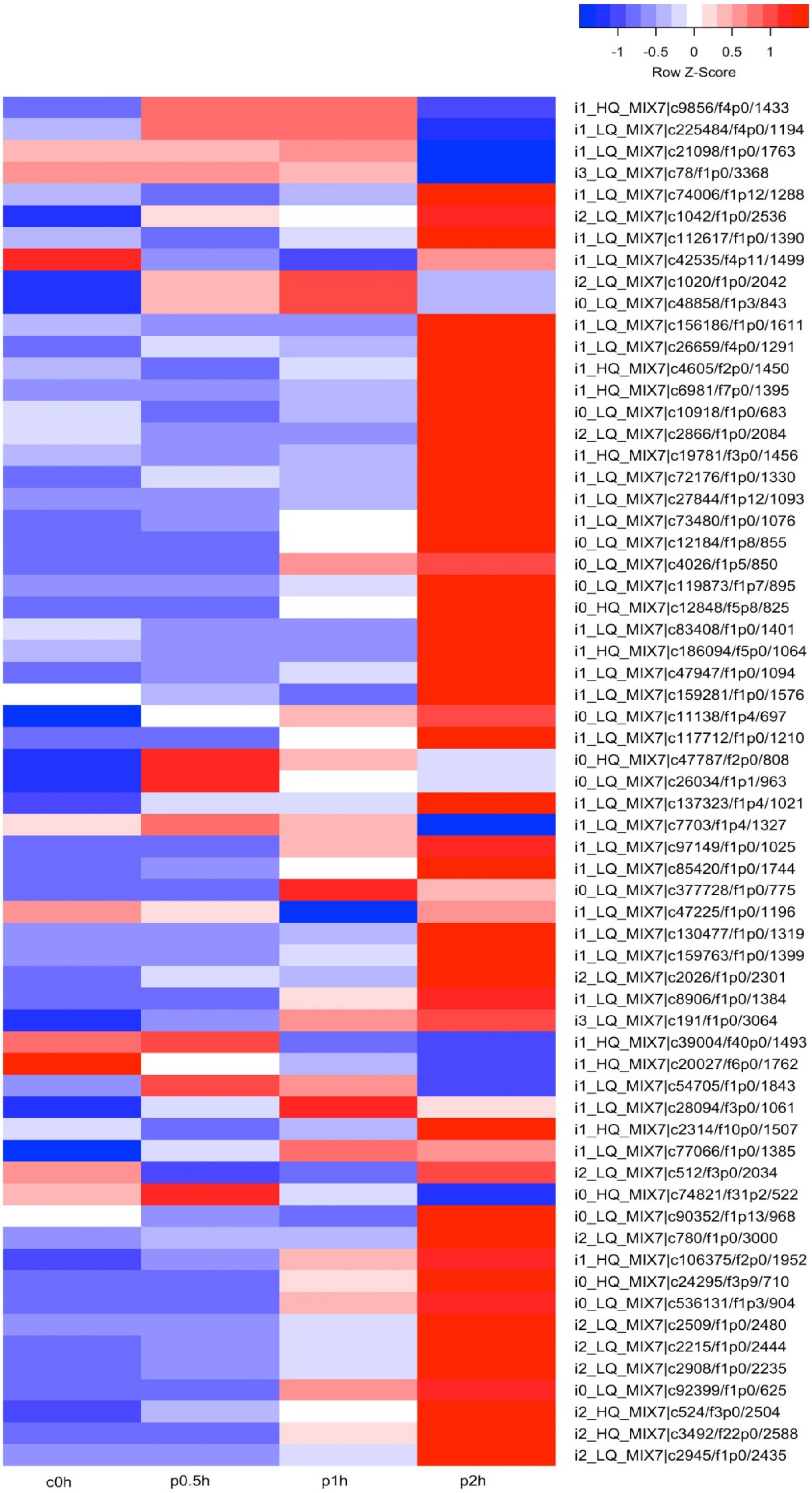

Figure S8, heat-map of protein processing in endoplasmic reticulum. Please refer to Fig. S2 for figure legends.

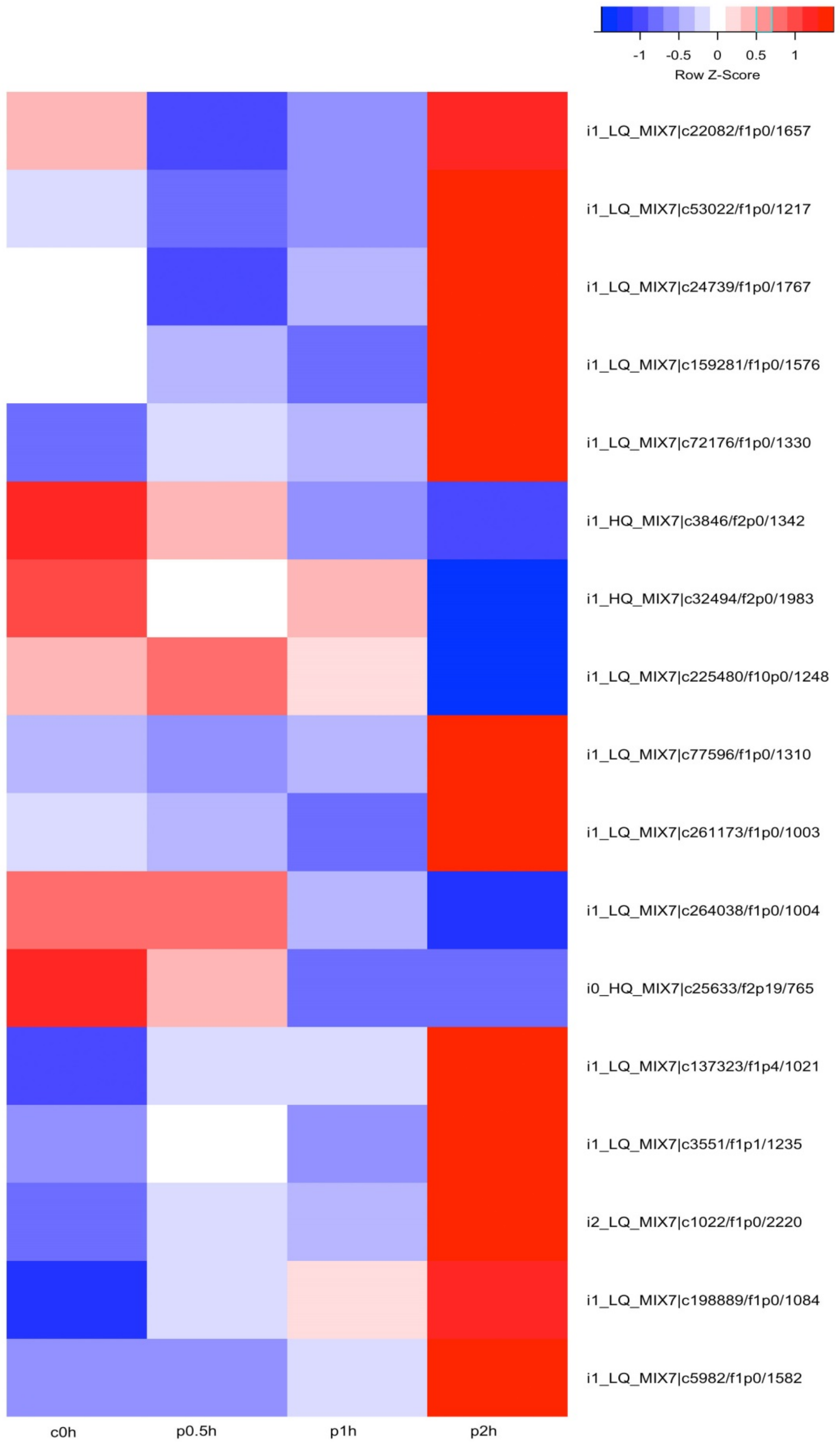

Figure S9, heat-map of ubiquitin mediated proteolysis. Please refer to Fig. S2 for figure legends.
