# Supplementary material for: Transcriptomic profiles of Dunaliella salina in response to hypersaline stress
Source: BMC Genomics. 2020 Feb 3;21:115. doi: 10.1186/s12864-020-6507-2 (PMC6998148; doi:10.1186/s12864-020-6507-2)
Supplement: Supplementary file 3 — Additional file 3. Confirmation of the expressions of the key genes by qPCR. [file 12864_2020_6507_MOESM3_ESM.pdf]

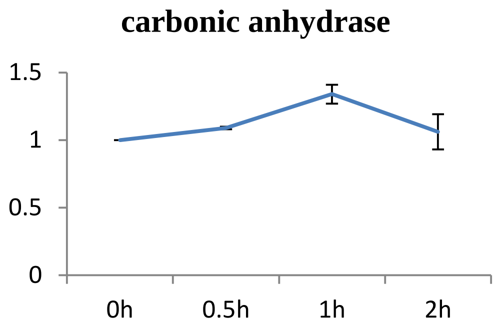
carbonic anhydrase
1.5
1
0.5
0
0h
0.5h
1h
2h

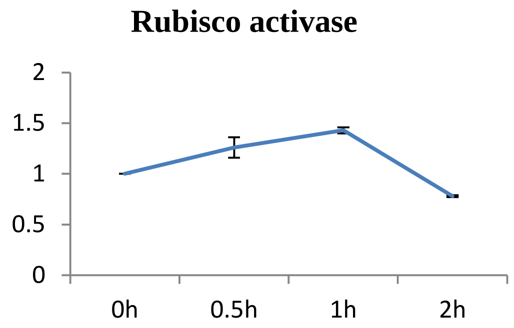
Rubisco activase
2
1.5
1
0.5
0
0h
0.5h
1h
2h

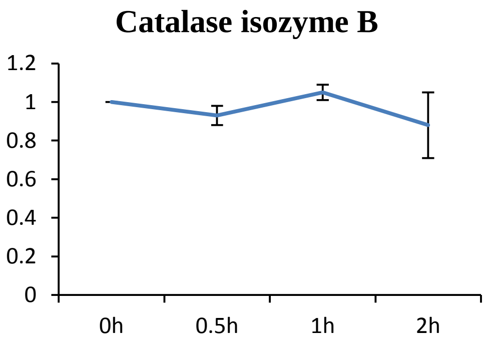
Catalase isozyme B
1.2
1
0.8
0.6
0.4
0.2
0
0h
0.5h
1h
2h

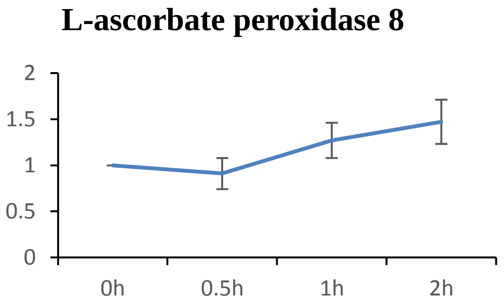
L-ascorbate peroxidase 8
2
1.5
1
0.5
0
0h
0.5h
1h
2h

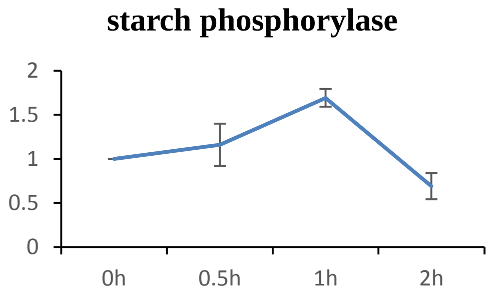
starch phosphorylase
2
1.5
1
0.5
0
0h
0.5h
1h
2h

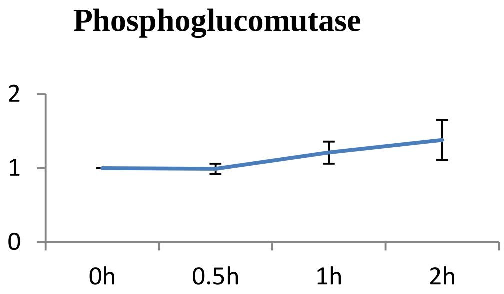
Phosphoglucomutase
2
1
0
0h
0.5h
1h
2h

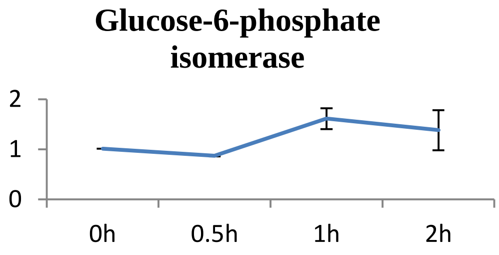
Glucose-6-phosphate isomerase
2
1
0
0h
0.5h
1h
2h

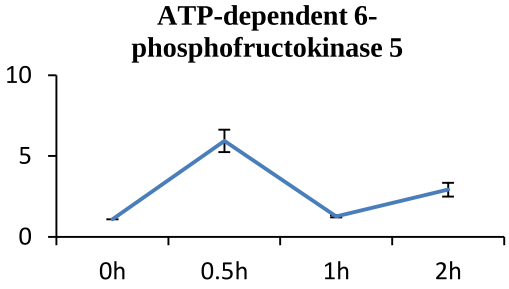
ATP-dependent 6-phosphofructokinase 5
10
5
0
0h
0.5h
1h
2h

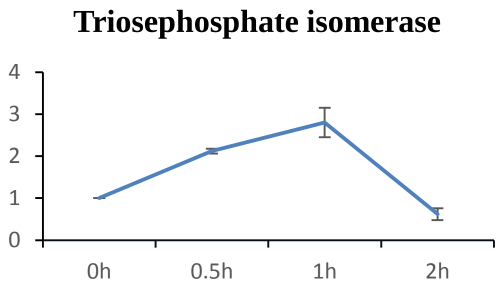
Triosephosphate isomerase
4
3
2
1
0
0h
0.5h
1h
2h

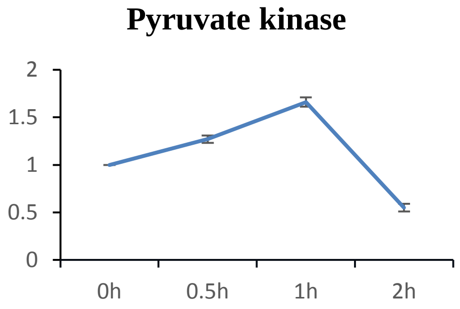
Pyruvate kinase
2
1.5
1
0.5
0
0h
0.5h
1h
2h

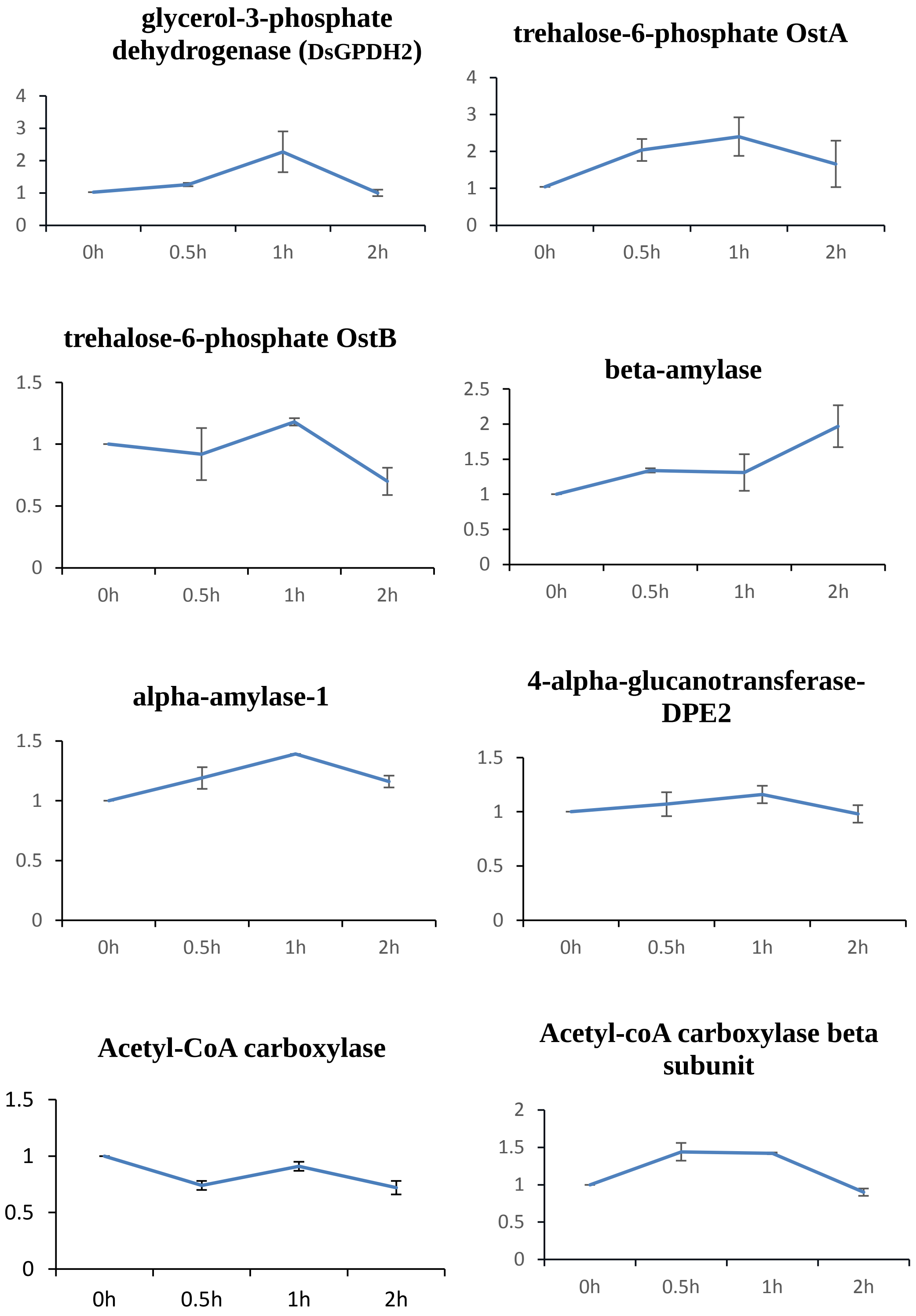
glycerol-3-phosphate dehydrogenase (DsGPDH2)
0 1 2 3 4
0h 0.5h 1h 2h
trehalose-6-phosphate OstA
0 1 2 3 4
0h 0.5h 1h 2h
trehalose-6-phosphate OstB
0 0.5 1 1.5
0h 0.5h 1h 2h
beta-amylase
0 0.5 1 1.5 2 2.5
0h 0.5h 1h 2h
alpha-amylase-1
0 0.5 1 1.5
0h 0.5h 1h 2h
4-alpha-glucanotransferase-DPE2
0 0.5 1 1.5
0h 0.5h 1h 2h
Acetyl-CoA carboxylase
0 0.5 1 1.5
0h 0.5h 1h 2h
Acetyl-coA carboxylase beta subunit
0 0.5 1 1.5 2
0h 0.5h 1h 2h

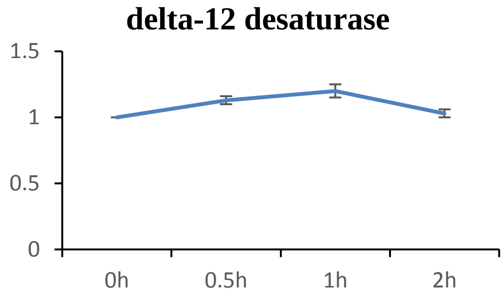
delta-12 desaturase
1.5
1
0.5
0
0h
0.5h
1h
2h

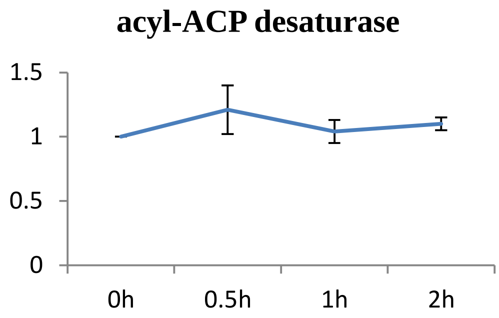
acyl-ACP desaturase
1.5
1
0.5
0
0h
0.5h
1h
2h

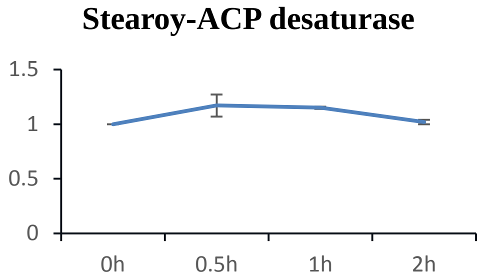
Stearoy-ACP desaturase
1.5
1
0.5
0
0h
0.5h
1h
2h

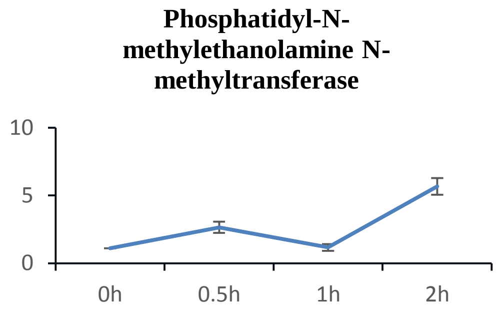
Phosphatidyl-N-methylethanolamine N-methyltransferase
10
5
0
0h
0.5h
1h
2h

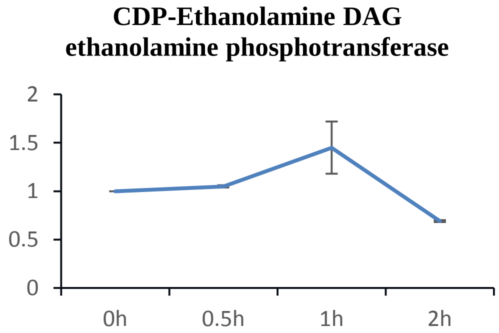
CDP-Ethanolamine DAG ethanolamine phosphotransferase
2
1.5
1
0.5
0
0h
0.5h
1h
2h

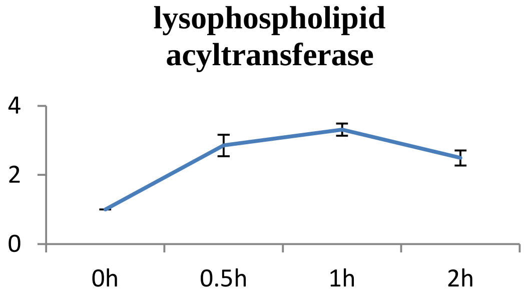
lysophospholipid acyltransferase
4
2
0
0h
0.5h
1h
2h

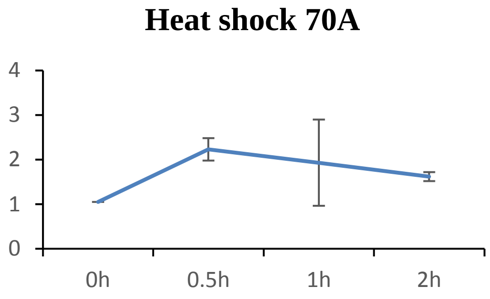
Heat shock 70A
4
3
2
1
0
0h
0.5h
1h
2h

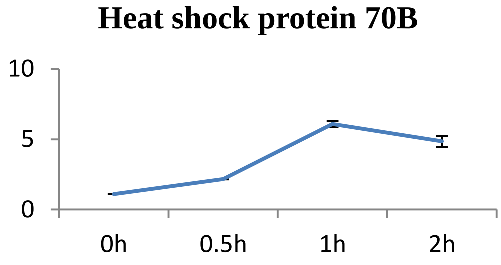
Heat shock protein 70B
10
5
0
0h
0.5h
1h
2h

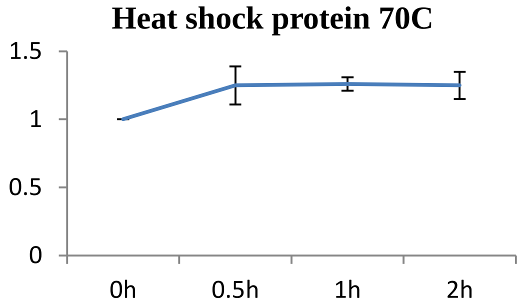
Heat shock protein 70C
1.5
1
0.5
0
0h
0.5h
1h
2h

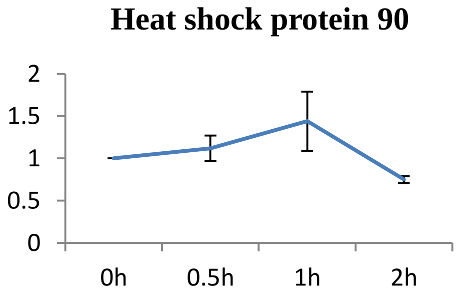
Heat shock protein 90
2
1.5
1
0.5
0
0h
0.5h
1h
2h

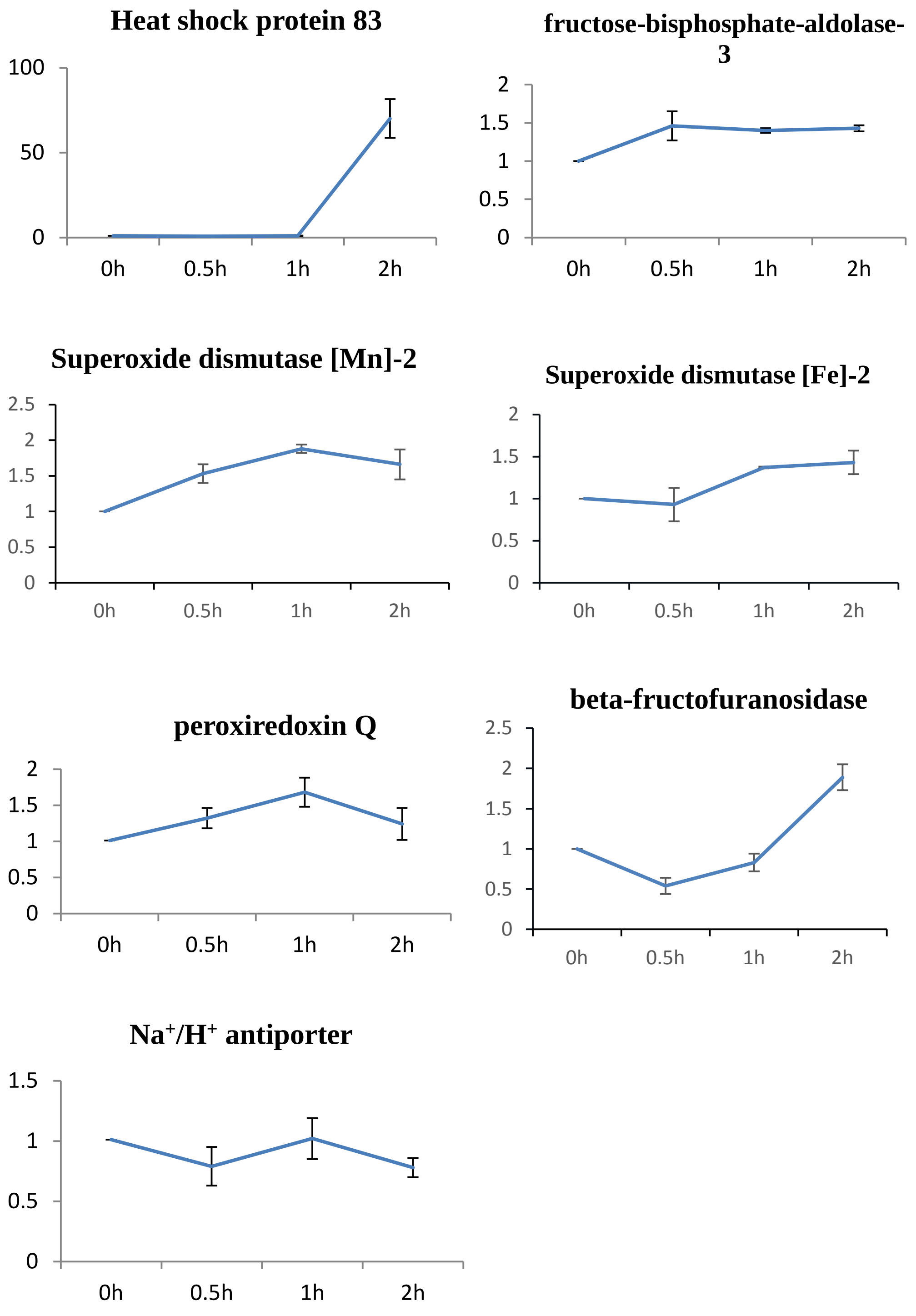

Additional file 3, confirmation of the expressions of the key genes by qPCR, the y axis represents the relative expression value; the x axis represents the salt stress time.

Primers used in qPCR

Gene name		Primer sequence
fructose-bisphosphate-aldolase-3	ps	AAGCGTTGGCCACTGGTTTAG
	pa	CACAGCACGCCATTTTGCA
Rubisco activase	ps	GGGCGCTGGAAGTCGATCGA
	pa	TTGTTGGTGCCCGCAGCTCC
carbonic anhydrase	ps	AAATTGCATCAGGTGCCATCA
	pa	AGCCAGCATATCCAAGCTCAGA
Phosphoglucomutase	ps	CCCTCAGACTCCGTTGCCA
	pa	CCGGTGGGGGTCTCAAAGTA
delta-12 desaturase	ps	CTCTGCCGATGCCGGTACA
	pa	GATGAGCACGGAACGCCAG
Pyruvate kinase	ps	TCAGCAAGGTTGAGAACCAGGA
	pa	TCATCTTTTGGGCCAGGAAGA
Stearoy-ACP desaturase	ps	TACATCCCTTCCCAGAAGACGC
	pa	GCTTTGCACGGGTTTGAGGTAG
Acetyl-coA carboxylase beta subunit	ps	TGCTGCACGGGATCCCTGT
	pa	CCTCCTGCGTGGCGTACTCA
Catalase isozyme B	ps	CGCACCATGCCTGGCTTT
	pa	CCTCGTCGTCCAGCAGGTTG
Na-H antiporter	ps	CCACCCAGGCCAGAATCTGTG
	pa	TGTCTGCCCTCGCACTGATCA
acyl-ACP desaturase	ps	GCCCCCATCATCCTCAACA
	pa	AGCACTTGTGCACAGGTGTCAG
Heat shock protein 83-1	ps	AGCGGAAATCAACCAACTGCT
	pa	ATGTGCATGTTGGGGTTGCT
Superoxide dismutase [Mn]-2	ps	GGGATGTTCACCGCCAAACT
	pa	TGCTCCATTGCAGCGTTGTAG
beta-fructofuranosidase	ps	AAGCCCAGCTTCCACCTGC
	pa	TCGTGCGGTGGTGCTGATAG
Superoxide dismutase [Fe]-2	ps	CTCACCCCCCCTCCTTATGGT
	pa	CGCCACCTTGACAAGCTTCAG
Acetyl-coA carboxylase beta subunit	ps	CATCCGGGCTATGGTTTCCT
	pa	TCCCTGGCTGAGTTCTTGTCAC
Phosphatidyl-N-methylethanolamine N-methyltransferase	ps	TGCCAAAATCCAGTCCCATACC
	pa	TGAACACGCTGGTCCACACTTC
ATP-dependent 6-phosphofructokinase 5	ps	CTGTGCTATTGCAGCGCGAA
	pa	TGAGGCAGGCCAAGATCTGAG
Heat shock protein 90-1	ps	CGTCATCCGCAGGCAGCTT
	pa	CGGCTGGAGAAGAAGCGCA
Heat shock protein 70C	ps	ATGTGAAGGACATCCTGCTGCT
	pa	TGTTGAGAAGACCTGGCCCTT
peroxiredoxin	ps	ATTAATCCACAGGGTGTGGTCG

	pa	GGCACACTTCTCCATGCTTCTC
peroxiredoxin Q	ps	GAACCCCTGGATGCACCAA
	pa	TTGCCCAGGCTGCATTCTC
lysophospholipid acyltransferase	ps	CGTGTCCGTGCCTCAGCTAC
	pa	GAAGGATGCCCGTGACCAG
Heat shock protein 70B	ps	GCACCATGTGCGGCAAGGAG
	pa	TGAGCTTGGTGGCCACACCG
Glucose-6-phosphate isomerase	ps	ACGGGCTCGACGTGACCAGA
	pa	TTCCACTCGCCACTGCGCA
starch phosphorylase	ps	CACCCCAACCAGTGGAACAA
	pa	GCTCGTCCTTGACGATCTCAGA
Heat shock 70A	ps	GAGACCGCTGGTGGTGTGATG
	pa	CGCTCACCCTCGTACACCTGA
beta-amylase	ps	CCTGAGTGGGGCTTGAGTGG
	pa	GCAGCAGTGAGGATGCGCT
glycerol-3-phosphate dehydrogenase (DsGPDH2)	ps	ATGCCCAGCATGCCTCCA
	pa	AGGCGTAGTGGCAGCAGCA
trehalose-6-phosphate OstA	ps	GAGGGCCAGACATGGAACACC
	pa	CGTCTCGATGCTGCTCCCAT
L-ascorbate peroxidase 8	ps	GAGGTGCCAACGGTGCCAT
	pa	TGGGAGACGTCAGGGTACTTGG
Triosephosphate isomerase	ps	AAGTTCTTTGTGGGCGGGAAC
	pa	TTGCAGTTGGAGTCGCTGACA
CDP-Ethanolamine DAG ethanolamine phosphotransferase	ps	GCCCTGGAGGGCTTGAAGGAGT
	pa	TACAACGCGGAGGCGAGGTAGG
alpha amylase-1	ps	AGGGAAGGCCTCCTGGTGTC
	pa	CCCTCAGGCAGGTTCTTCCA
trehalose-6-phosphate OstB	ps	CCACAGATGTGGATGGCACAC
	pa	GGTTGAGCTGCGAGAACACCT
4-alpha-glucanotransferase-DPE2	ps	ACAAGTGGTGGAGGAGAAGGCT
	pa	CGGCTGTCAAGCTCATGCTT
